# Optimizing strength of directly recycled aluminum chip-based parts through a hybrid RSM-GA-ANN approach in sustainable hot forging

**DOI:** 10.1371/journal.pone.0300504

**Published:** 2024-03-14

**Authors:** Yahya M. Altharan, Shazarel Shamsudin, Mohd Amri Lajis, Sami Al-Alimi, Nur Kamilah Yusuf, Nayef Abdulwahab Mohammed Alduais, Atef M. Ghaleb, Wenbin Zhou

**Affiliations:** 1 Sustainable Manufacturing and Recycling Technology, Advanced Manufacturing and Materials Center (SMART‐AMMC), Universiti Tun Hussein Onn Malaysia, Parit Raja, Malaysia; 2 Faculty of Computer Science and Information Technology (FSKTM), Universiti Tun Hussein Onn Malaysia (UTHM), Parit Raja, Batu Pahat, Johor, Malaysia; 3 Department of Industrial Engineering, College of Engineering Alfaisal University, Riyadh, Saudi Arabia; 4 School of Science and Engineering, University of Dundee, Dundee, United Kingdom; Industrial University of Ho Chi Minh City, VIET NAM

## Abstract

Direct recycling of aluminum waste is crucial in sustainable manufacturing to mitigate environmental impact and conserve resources. This work was carried out to study the application of hot press forging (HPF) in recycling AA6061 aluminum chip waste, aiming to optimize operating factors using Response Surface Methodology (RSM), Artificial Neural Network (ANN) and Genetic algorithm (GA) strategy to maximize the strength of recycled parts. The experimental runs were designed using Full factorial and RSM via Minitab 21 software. RSM-ANN models were employed to examine the effect of factors and their interactions on response and to predict output, while GA-RSM and GA-ANN were used for optimization. The chips of different morphology were cold compressed into billet form and then hot forged. The effect of varying forging temperature (Tp, 450–550°C), holding time (HT, 60–120 minutes), and chip surface area to volume ratio (A_S_:V, 15.4–52.6 mm^2^/mm^3^) on ultimate tensile strength (UTS) was examined. Maximum UTS (237.4 MPa) was achieved at 550°C, 120 minutes and 15.4 mm^2^/mm^3^ of chip’s A_S_: V. The Tp had the largest contributing effect ratio on the UTS, followed by HT and A_S_:V according to ANOVA analysis. The proposed optimization process suggested 550°C, 60 minutes, and 15.4 mm^2^ as the optimal condition yielding the maximum UTS. The developed models’ evaluation results showed that ANN (with MSE = 1.48%) outperformed RSM model. Overall, the study promotes sustainable production by demonstrating the potential of integrating RSM and ML to optimize complex manufacturing processes and improve product quality.

## 1. Introduction

Recycling material waste reduces landfills and provides a viable economic solution. In recent years, there has been a notable surge in focus on aluminum waste recycling, driven by its potential to achieve resource conservation, reduce energy consumption, and promote environmental sustainability [1–3]. Specifically, solid-state (meltless-based) recycling techniques have emerged as a promising pathway for converting discarded material into valuable components. Solid-state recycling (SSR) is a preferred choice as it is an energy-efficient and eco-friendly technique, particularly for handling the scrap chips of aluminium. This method can convert the scraps directly into semi-finished and finished products of superior mechanical properties, eliminating the need for remelting. Several SSR processes, including hot forging, extrusion, high-pressure torsion, and friction stir welding, have demonstrated the ability to produce aluminum chip-based products with high performance [4–6]. Utilizing SSR recycling processes to efficiently recycle aluminum chip waste into functional parts reduces material waste and energy consumption compared to the remelting method [5, 7, 8].

Among SSR techniques, hot press forging (HPF) was found to be an efficient way of promoting intermetallic bonding in aluminum chip recycling [9–11]. In the HPF technique, aluminum chip waste is directly consolidated by heating and high pressure. When metal is subjected to high temperatures and pressure, its microstructure becomes more malleable, formable, and easily shaped through a die [12]. This technique has several advantages over other SSR techniques, including strength improvement, hardness, and ductility [13]. Multiple studies have reported that HPF significantly improves the mechanical properties of recycled aluminum parts [5, 11, 14–17]. Forged parts exhibited 20% and 40% higher yield strength (YS) and ultimate tensile strength (UTS) than as-cast material at 500–550°C and 1 s-1 strain rate [18]. The properties of chip-based forged material are substantially affected by operating temperatures. Several studies have shown that 530–550°C forging temperature and 120 min holding time resulted in higher UTS and hardness [10, 11, 19, 20]. Nevertheless, the morphology of the aluminum chip, including surface roughness, size, and shape structure, influenced the direct recycling of aluminum alloy 6061 (AA6061) waste [21, 22].

The strength improvement of AA6061 chip-based recycled material is a major challenge in HPF direct recycling methods. Another challenge is determining optimal processing parameters and machining chip morphology to achieve the desired strength of recycled material [21, 22]. Modeling and optimizing the processing parameters and chip morphology can significantly contribute to improving the chip-based recycled strength. Recently, there has been a growing interest in utilizing advanced techniques, such as Response Surface Methodology (RSM) and Machine Learning (ML), to model and optimize the performance of manufacturing processes [23–27]. The aluminum waste recycling parameters have been modeled using RSM [10, 11, 28, 29] and ANN [26, 30–34]. Moghri et al. [35] reported that RSM and genetic algorithm effectively identified the optimum process variables for maximum tensile modulus and tensile strength of PA-6/clay nanocomposite. Alateyah et al. [36] concluded that the hybrid RSM-GA method efficiently enhanced the hardness, ultimate tensile strength, and electrical conductivity of pure Cu. Yeniay [37] suggested that the integrated GA-RSM can improve optimization outcomes better than conventional gradient-based approaches. Praga-Alejo et al. [38] reported that ANN-GA performed well in identifying the ideal parameters for process response Compared to RSM. In [39], the age hardening process of aluminum alloy A356/cow horn particulate composite was modeled using RSM and ANN and optimized by a simulated annealing (SA) algorithm. The developed ANN model outperformed the RSM model in age hardening data prediction, with a correlation coefficient (R^2^) of 0.9921. With a 1.2% relative error, SA-NN optimization results matched experimental values. Zulfiqar et al. [40] adopted RSM optimization and ANN modeling for photocatalytic degradation of acid orange 7 (AO7) in wastewater treatment using TiO2-P25 nanoparticles (TNPs). ANNs accurately predicted AO7 degradation with a high R^2^ value, indicating a strong correlation with experimental data. The application of RSM, ANN, and GA for modeling and optimizing processing parameters proved highly effective, with remarkably accurate results.

This study explores the effectiveness of this innovative RSM-ML approach in modeling and optimizing processing parameters (i.e., forging temperature, holding time, and chip morphology) in HPF to achieve the desired strength of the recycled parts. This approach is absent from most previous studies of recycling aluminum chips using HPF. The ML was based on the ANN and GA algorithm. ANN is a computational configuration used to simulate the biological neural system concerning the information-processing capabilities of neurons. It exhibits a high degree of parallelism, which utilizes numerous interconnected units to process information [41]. ANN consists of several neurons as processing units, categorized as input, hidden layers and output. Each neuron contains three basic parts: weights, bias, and transfer function. Typically, the input is weighted, and the bias is added to its value before passing through the activation function [42]. The architecture and algorithm of the network determine its prediction accuracy and learning rate [43]. Among the various ANN algorithms and activation functions, backpropagation and the ReLU function are deemed more appropriate for processing regression data [44–47]. The purlin linear function is used for numerical prediction problems where the output value is continuous, and the objective is to predict the output value. The ANN-based approach was employed to model the relationship between input forging process parameters and output UTS response to identify data patterns.

The RSM-based approach is a powerful statistical tool widely employed for parameter optimization, experimental design, model fitting, and validation. It enables the development of a polynomial regression equation to model the relationship between the processing parameters and the desired response [48]. With its capacity to model quadratic, linear and interaction effects, RSM was chosen to investigate factors influencing response. GA is a category of numerical and combinational optimizers that are particularly helpful for solving complex linear and nonlinear problems [49–51]. It was introduced by John Holland in the 1970s based on Darwin’s theory of evolution. This algorithm imitates life’s evolution process by modifying a population of individual solutions in which only the fittest survive through mutation, selection, and crossover. Each proposed solution has a set of genes or properties that can mutate or change until the optimal solution is captured. The hybrid RSM-ML combines the strength of both methods, leveraging RSM’s statistical rigor and the ML algorithms’ predictive power. The prediction performance of RSM and ANN models is evaluated based on processing parameters.

The effect of processing parameters was investigated based on low, medium and high levels. The operating temperature levels were 450, 500, and 550°C, and holding times were 60, 90, and 120 minutes. In addition, three different chip types with 15.4, 34 and 52.6 mm^2^/mm^3^ surface-area-to-volume ratio (A_S_: V). Small, medium, and large aluminum chips with measured A_S_: V were recycled to evaluate the impact of the oxidation on chip welding. The oxide is typically formed on the surface of chips due to exposure to air during machining and recycling processes. The variation in oxide content is proportional to the chip’s surface area. This oxidation positively influences the properties of the recycled material with refined grain structure [52]. However, the excessive and uneven distribution of oxide precipitate in recycled samples may negatively impact elongation to failure and tensile strength [7, 53]. Consequently, this work aims to comprehensively investigate the effect of chip morphology (size and A_S_:V) and operational parameters on UTS using an integrated RSM-GA-ANN strategy approach. The main objective is to attain the maximum UTS of chip-based recycled parts.

To accomplish this goal, an experimental run was designed using a full factorial and Central Composite Design (CCD) via Minitab 21 software to minimize costs and save time. This design enables the creation of a diverse combination of processing parameters with different levels [48]. By systematically varying the process, the desired number of experiments are conducted. The collected dataset of experimental results was statistically analyzed using Analysis of Variance (ANOVA) to reveal the variations and input-output relationships. This analysis provides a basis for understanding the effect of single and interactive parameters on the UTS of the recycled parts.

The novel contributions of this study lie in investigating the effect of aluminum machining chip morphology (size and surface area) on the strength of HPF recycled parts by employing an innovative hybrid RSM-ANN-GA approach. This study uniquely models and optimizes the relationship between chip formation and the HPFed recycled part’s UTS. This contributes to the advancement of the recycling process and provides a better comprehension of the complex relationship between chip morphology and the recycled products’ strength. The outcomes may offer valuable insights and practical solutions for manufacturing industries to estimate the strength of chip-based recycled products by choosing specific chip size and controlling process parameters using established model.

## 2. Materials and methods

**Fig 1:**
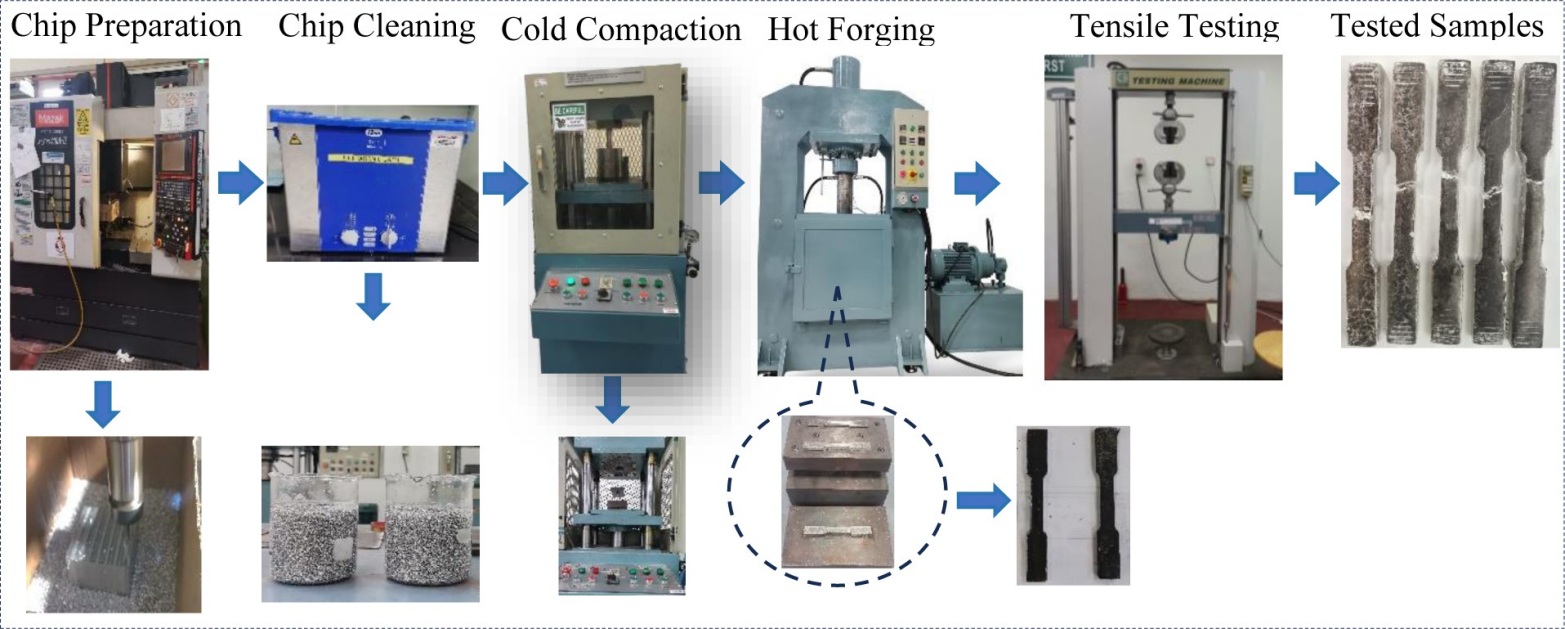
Experimental flow process.

This section presents the materials, methods, and experimental steps, as illustrated in process flow in Fig 1. The experimental setup, data collection, and analysis techniques are described, facilitating a comprehensive understanding of the research methodology. The process of developing the predictive models and optimization is explained in detail. In this work, three different sizes of chips were produced by machining AA6061-T6 aluminum block using a CNC Mazak NEXUS 410A-II VMC milling machine. The machining parameters were manipulated to generate the desired chip size, as outlined in Table 1. The chips were precision-machined to ensure an average length of 4 ± 1mm, as recommended by Gronostajski et al. [54].

**Table 1:** Machining parameters and average size of chips.

Chip Type	Parameters			Average chip size (mm)			
	Cutting Speed (mm/min)	Depth of Cut (mm)	Feed Rate, (mm/rev)	Length	Width	Thickness	Average A_S_: V (mm^2^/mm^3^)
Small	1100	0.5	0.04	4.8	0.54	0.0380	52.6
Medium	1100	1	0.1	3.6	1.17	0.0628	34.0
Large	1100	1.5	0.2	4.27	1.68	0.1450	15.4

The originality of the used Al6061 block was verified through a chemical composition examination utilizing a scanning electron microscope with energy dispersive X-ray spectroscopy (SEM-EDS), as shown in Table 2.

**Table 2:** The chemical composition of AA6061.

Element	*Si*	*Fe*	*Cu*	*Mn*	*Mg*	*Zn*	*Cr*	*Ni*	*Ti*
(wt. %)	0.4–0.8	0.7 Max.	0.15–0.40	0.15Max.	0.8–1.2	0.25 Max	0.04–0.35	0.06	0.15 Max.

**Fig 2:**
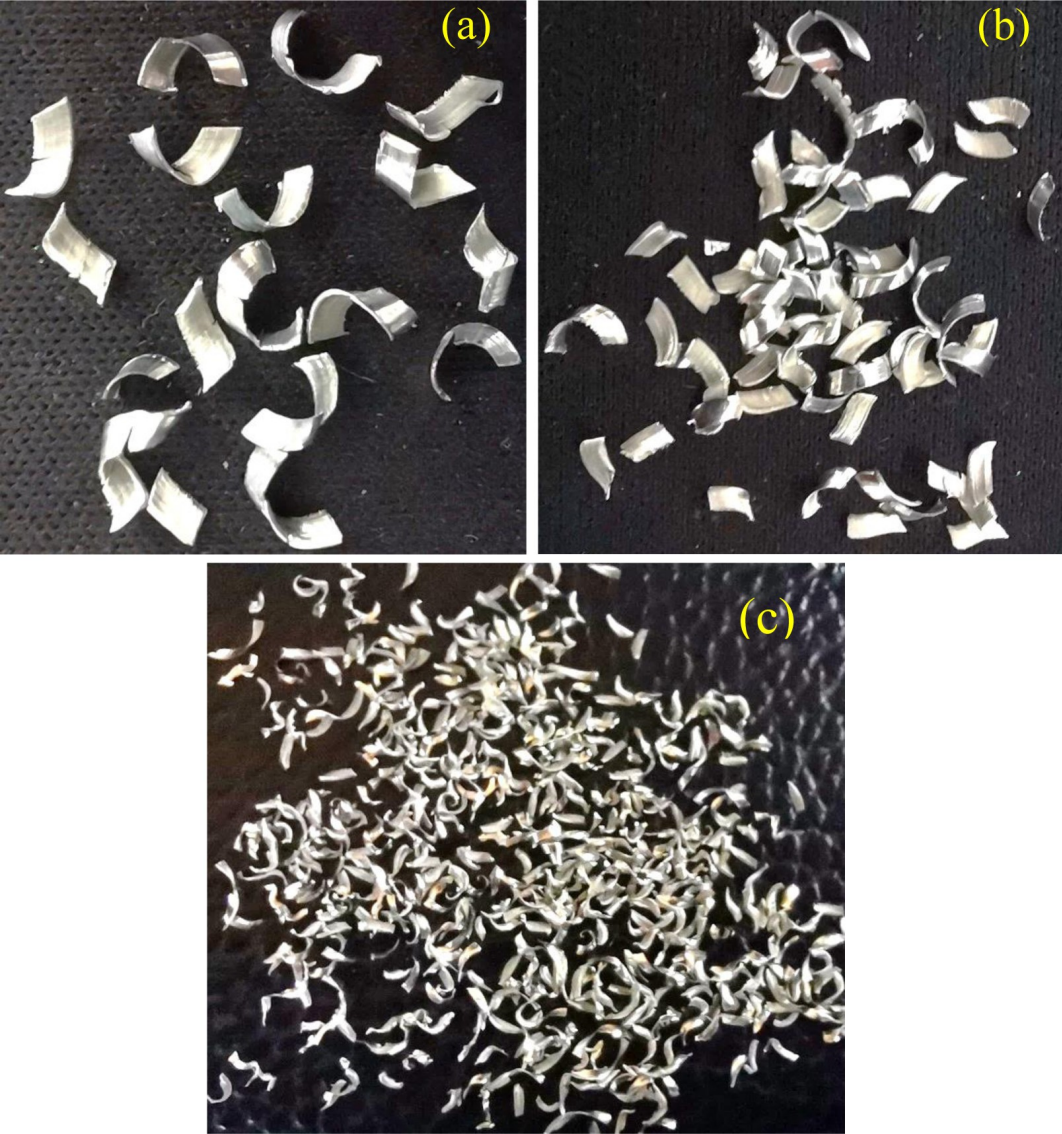
Aluminum machined chips; (a) large, (b) medium and (c) small size.


The different chips were prepared to investigate the effect of their formation on the recycled part’s strength. The large-sized chips were segmented and curled, while medium-sized and small-sized chips were characterized by fragmented-discontinuous and thinner-spiral shapes, respectively, as depicted in Fig 2. The average size of the chip particles was measured by a Dino-lite microscope with a digital Nikon MM-60 camera. The chips produced were in various forms and surface areas. The chip’s A_S_: V depends on its size, where small chips have a large surface area and vice versa. The chips with a larger A_S_: V tend to have more oxide formation. The A_S_: V is a critical parameter in chip welding due to the potential accumulation of oxide. Assuming that the machined chips are in cubic shape, Eq (1) was utilized to estimate the average A_S_: V of the chips [55].

$$S=\frac{2(lw+wt+tl)}{lwt}$$ (1)

Where *l*, *w* and *t* are the length, width, and thickness, respectively.

### 2.1 Design of experiment

**Fig 3:**
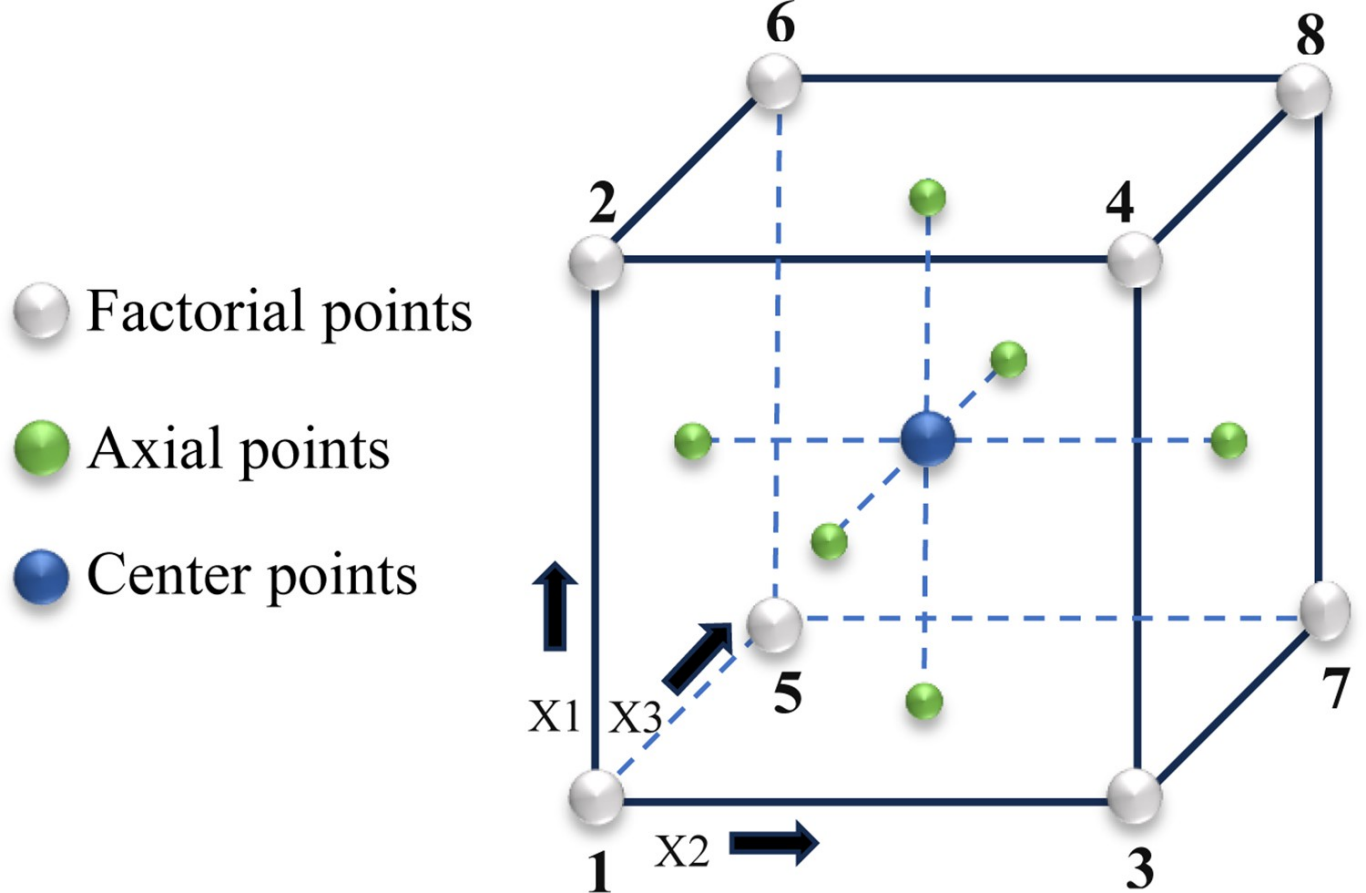
The 2^3^ full factorial and face-centred central composite design [58].

The experimental runs were designed using two-level full factorial and RSM Central Composite design (CCD) via Minitab statistical software ver. 21. A factorial and CCD face-centered design enables the experimenter to evaluate the interaction between independent factors and dependent responses systematically [56]. The two-level full factorial design is a widely utilized experimental methodology within the industry [57]. This work’s full factorial design involved three factors, each with two levels (2^3^), two replications, and three center points. The CCD face-centred design is an extension of the factorial design that incorporates both factorial and axial points, with a standardized alpha (α) value of 1. In this design, the axial point is located on the centres of the cube faces, as shown in Fig 3. The core concept of CCD is to investigate the response surface systematically to comprehend the variables’ interaction and optimize the process. The alpha (α) level controls the distance of axial points from the centre of the experimental design (determines how far the axial points are from the centre). The smaller the alpha level, the closer the axial points are to the center.

The cubic box in Fig 3 graphically represents the 2^3^-face-centered design. The arrows in the box denote the direction of the factor increasing and numbers ’1 to 8’ in the corners indicate the "Standard Order" of runs. The CCD axial points are orthogonal at a distance of ±1 (alpha level) from the center point along each axis [58].

The 2^3^ face-cantered design matrix with three factors (X1, X2, X3) is presented in Table 3. The low setting is denoted by (-1) and the high setting with (+1), while the centre point (0) is taken at the midpoint between the low (-1) and high (+1) levels of each factor.

**Table 3:** The 23 face-centred design matrix.

Run Order	Centre points	Blocks	Input Factors		
			X1	X2	X3
1	1	1	-1	-1	-1
2	1	1	+1	-1	-1
3	1	1	-1	+1	-1
4	1	1	+1	+1	-1
5	1	1	-1	-1	+1
6	1	1	+1	-1	+1
7	1	1	-1	+1	+1
8	1	1	+1	+1	+1
9	1	1	-1	-1	-1
10	1	1	+1	-1	-1
11	1	1	-1	+1	-1
12	1	1	+1	+1	-1
13	1	1	-1	-1	+1
14	1	1	+1	-1	+1
15	1	1	-1	+1	+1
16	1	1	+1	+1	+1
17	0	1	0	0	0
18	0	1	0	0	0
19	0	1	0	0	0
20	-1	2	-1	0	0
21	-1	2	+1	0	0
22	-1	2	0	-1	0
23	-1	2	0	+1	0
24	-1	2	0	0	-1
25	-1	2	0	0	+1
26	0	2	0	0	0
27	0	2	0	0	0

The two-level 2^3^ design scheme of the uncontrolled processing parameters is listed in Table 4. The controlled parameters, including pressure (p) and homogenization time (t_h_) were fixed at 35 tons and 45 minutes, respectively, as confirmed by Kamilah et al. [59].

**Table 4:** The design scheme of the process parameters (uncontrolled variables).

Factor symbol	Parameter	Levels		
		Low (−1)	Center (0)	High (+1)
*T*_*p*_	Operating temperature, (°C)	450	500	550
*HT*	Holding Time (minutes)	60	90	120
*CSA*	Chip Surface Area (mm^2^)	15.4	34	52.6

Nineteen experiments were designed with full factorial design. Additionally, eight axial face-centered runs using CCD were added because the curvature effect was found to be statistically significant upon ANOVA analysis of the 19 experiments. A total of 27 runs were involved in the experimental study, as shown in Table 5.

**Table 5:** Experimental design runs with UTS test results.

Run Order	Centre Point	Input Factors			Response	Design Runs
		Temp. (Tp)°C	Holding Time (HT) min	Chip Surface Area (CSA) mm^2^	UTS	
1	1	450	60	15.4	28.500	Full Factorial Design with 2 Replications and 3 Center Points
2	1	550	60	15.4	191.70	
3	1	450	120	15.4	58.200	
4	1	550	120	15.4	235.30	
5	1	450	60	52.6	20.700	
6	1	550	60	52.6	172.01	
7	1	450	120	52.6	42.300	
8	1	550	120	52.6	206.70	
9	1	450	60	15.4	26.900	
10	1	550	60	15.4	193.80	
11	1	450	120	15.4	54.800	
12	1	550	120	15.4	237.40	
13	1	450	60	52.6	18.300	
14	1	550	60	52.6	171.40	
15	1	450	120	52.6	43.700	
16	1	550	120	52.6	210.60	
17	0	500	90	34.0	154.20	
18	0	500	90	34.0	152.93	
19	0	500	90	34.0	155.30	
20	-1	450	90	34.0	33.010	Additional Axial Points-RSM Runs with 2 Center Points
21	-1	550	90	34.0	202.66	
22	-1	500	60	34.0	135.00	
23	-1	500	120	34.0	164.60	
24	-1	500	90	15.4	161.40	
25	-1	500	90	52.6	140.83	
26	0	500	90	34.0	151.24	
27	0	500	90	34.0	154.97	

### 2.2 Hot Press Forging Process (HPF)

**Fig 4:**
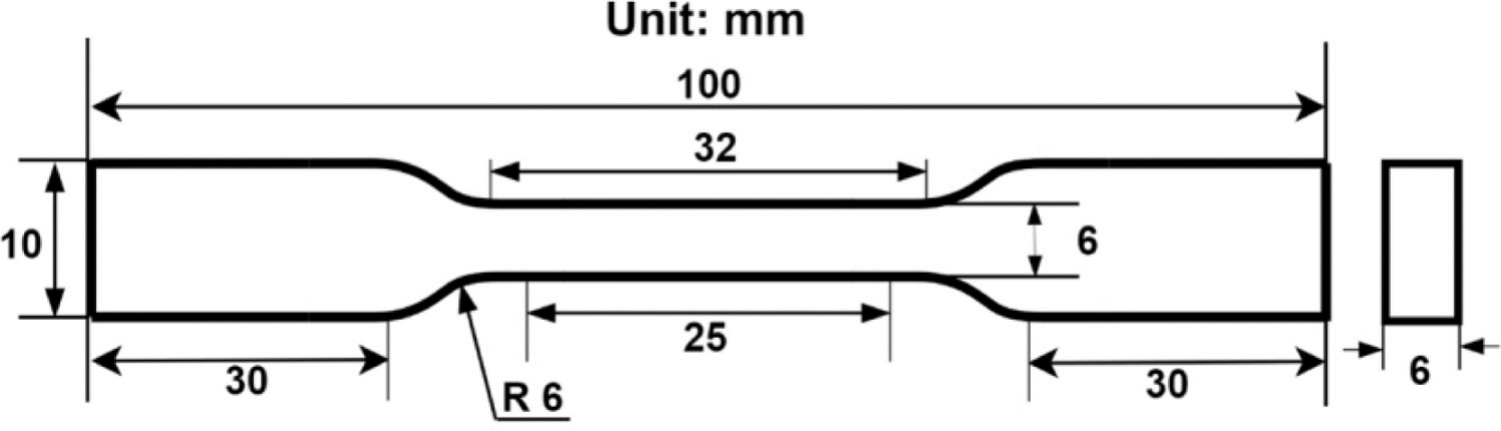
Plate-type tension test specimen (ASTM E8M).

**Fig 5:**
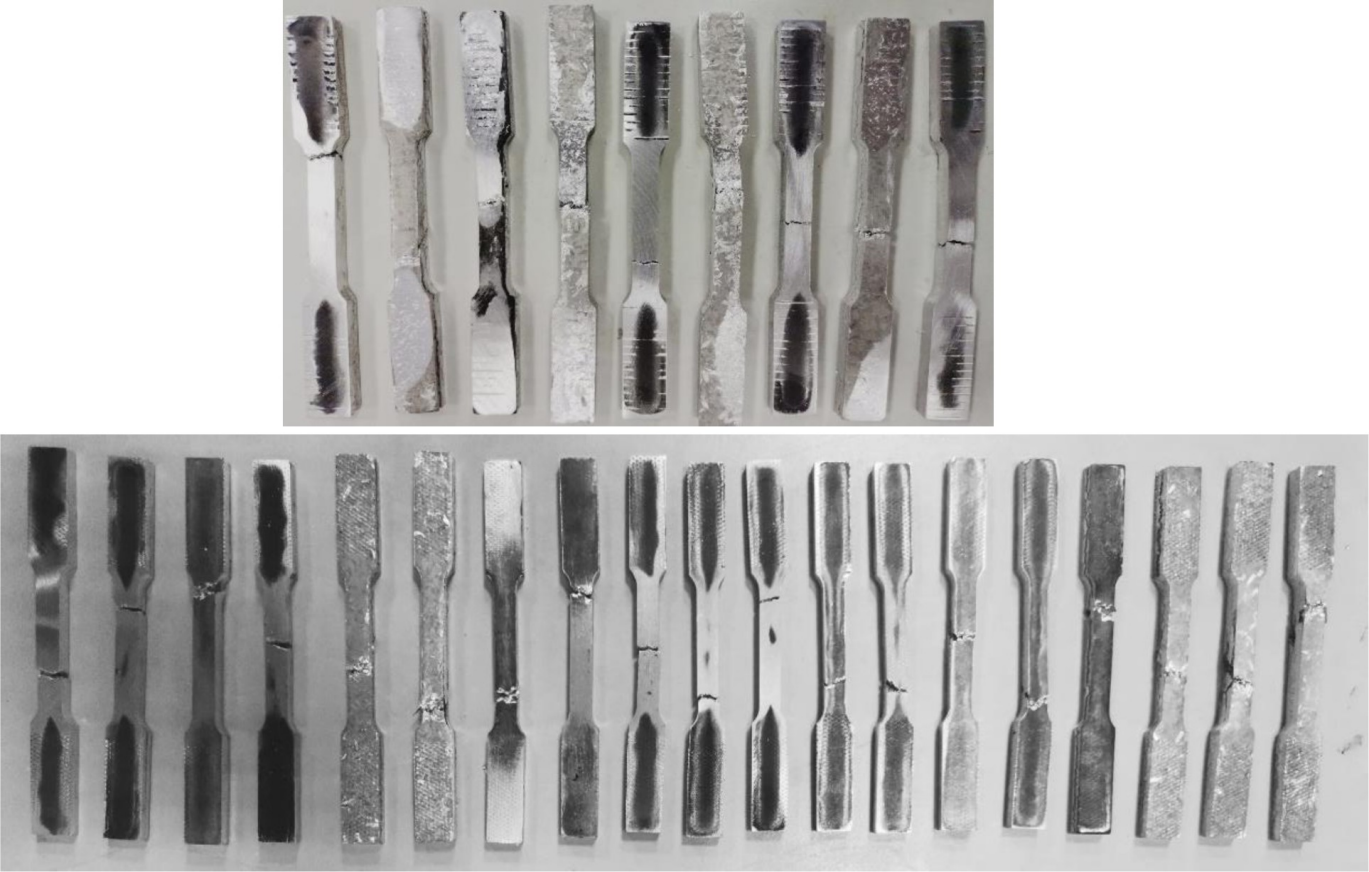
Recycled samples after tensile test.

Hot forging is a popular solid-state recycling method, consisting of three main procedures: chip cleaning and drying, subsequently cold compaction into a billet, and finally, hot forging. The machined chips were cleaned and degreased in Aceton A.R solution for 30 minutes and an Elmasonic S 60 H bath as per the ASTM G131-96 standard, and then thoroughly dried in an oven at 100°C for 30 minutes. Prior to cold compaction and hot forging, 12g of chips were weighed to ensure the prepared sample size conformed to the ASTM E8M standard [60]. The chip was then placed into a dog bone-shaped die and compacted into billet form under 35 tons of pressure at room temperature. Afterward, the billet die was preheated for 45 minutes of homogenization time in the forging machine, followed by the forging process following the run order in Table 5. The proposed forging temperatures (Tp) were 450–550°C between the recrystallization and solidus points, while the holding time was between 60–120 min. The exact geometric dimensions of produced specimens are based on ASTM E8M, as shown in Fig 4. The HPFed produced samples after tensile strength testing are depicted in Fig 5.

### 2.3 Modeling and optimization process

**Fig 6:**
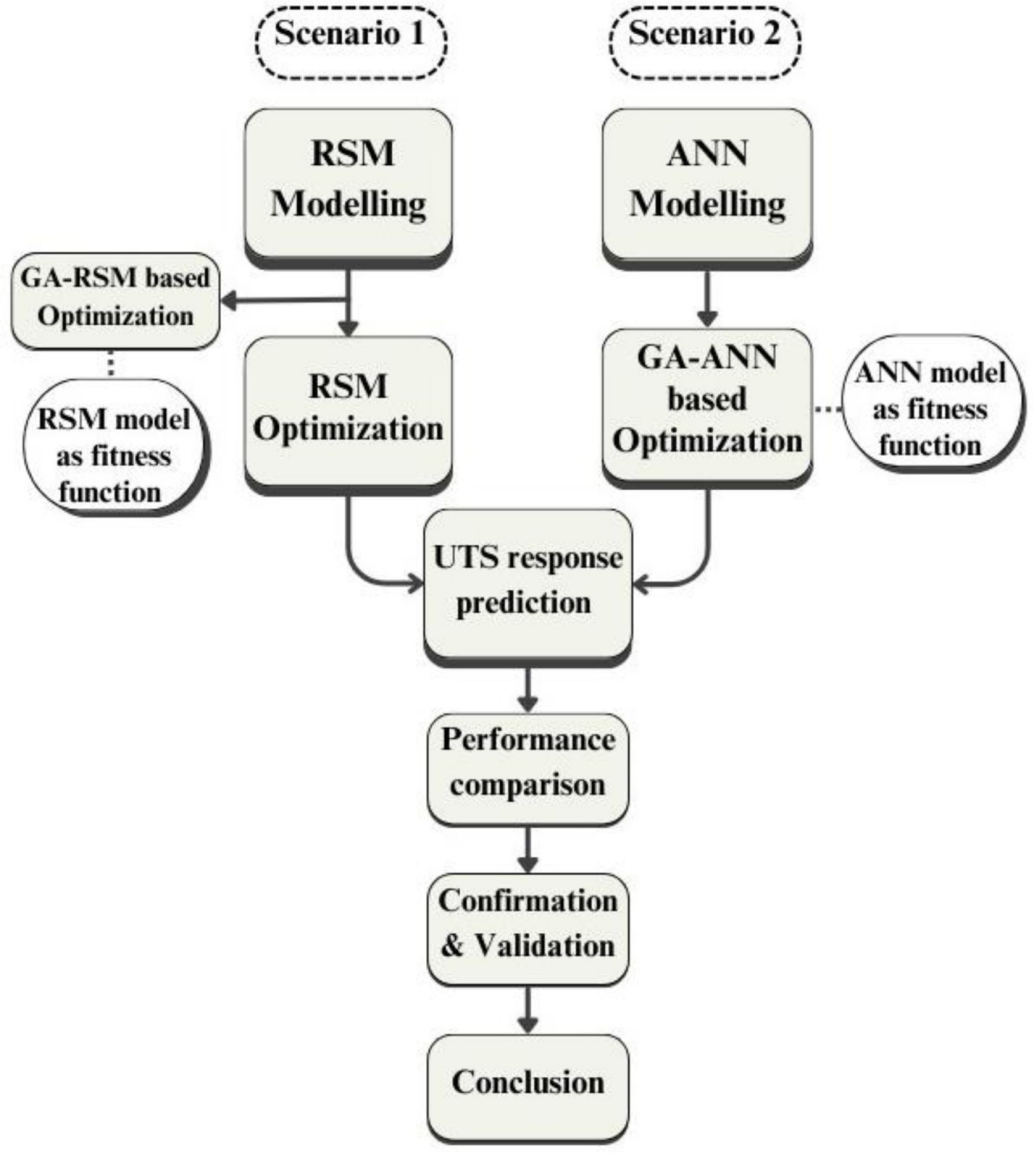
Block diagram of modeling and optimization processes flow.

The RSM and ANN models were adopted to predict the UTS of recycled samples based on different sets of processing parameters. Fig 6 presents the modeling and optimization processes flow. RSM and ANN models were developed for optimization and prediction of UTS response. The RSM quadratic polynomial regression model is an algebraic representation describing the relationship between the input terms and output response to estimate each factor effect and their interaction. RSM regression equation is derived by Minitab software based on experimental data analysis to assess the relationship between input factors and output response.

The three-factor interaction of the full model is expressed in Eq (2) as follows:

$$Y=\beta_{0}+\sum_{i=1}^{n}\beta_{i}\;x_{i}+\sum_{i=1}^{n}\beta_{ii}\;x_{i}^{2}\pm \varepsilon$$ (2)

Where *Y* is response, *β*_0_ is the constant, *x*_*i*_ is the coded level of the factor, and *ε* is the experimental error.

**Fig 7:**
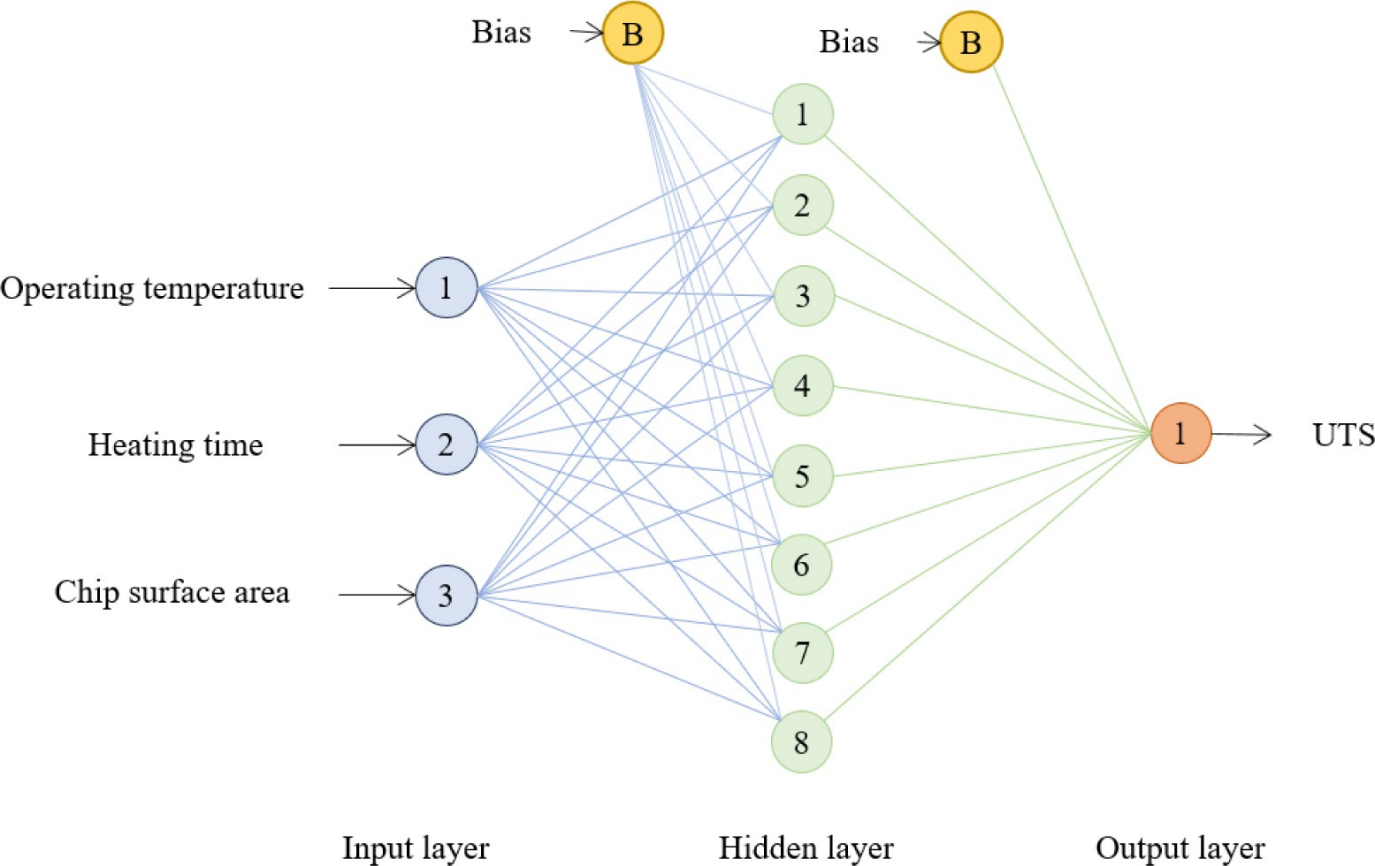
The ANN configuration for UTS prediction.

The ANN model was developed to predict tensile strength for similar combinations of processing factors. Based on the training data, the ANN model can approximate the relationship between the input factors and the output response. The input factors (forging temperature, pressing time and A_S_: V of chip) were set in the input layer neurons, while the UTS response was the output layer. The ANN architecture employed in this investigation is depicted in Fig 7. The ANN was trained using an experimental dataset (27 samples) with ratios of 70%, 15% and 15% for training, testing and validation, respectively. Unlike RSM, ANN modeling requires a comprehensive set of experimental data.

Nevertheless, ANN can model a small database with a strong statistical correlation between variables using techniques such as leave-one-out cross-validation (LOOCV) [61]. To prevent overfitting in training a limited dataset of 27 points, LOOCV was employed to improve the performance of the NN model. In LOOCV, the model is repeatedly trained using all but one data point left out for testing. This is repeated for each data point within the dataset to ensure that each data point is used for testing exactly once, to estimate the model’s generalization performance and to mitigate the impact of overfitting on a small dataset [62]. The regularization of 0.5 was also adopted to prevent overfitting by adding a penalty term to the error function during training. Regularization is a function that penalizes large weights. By penalizing large weights, regularization encourages the model to have simpler weight configurations and reduces the risk of overfitting [63, 64].

**Fig 8:**
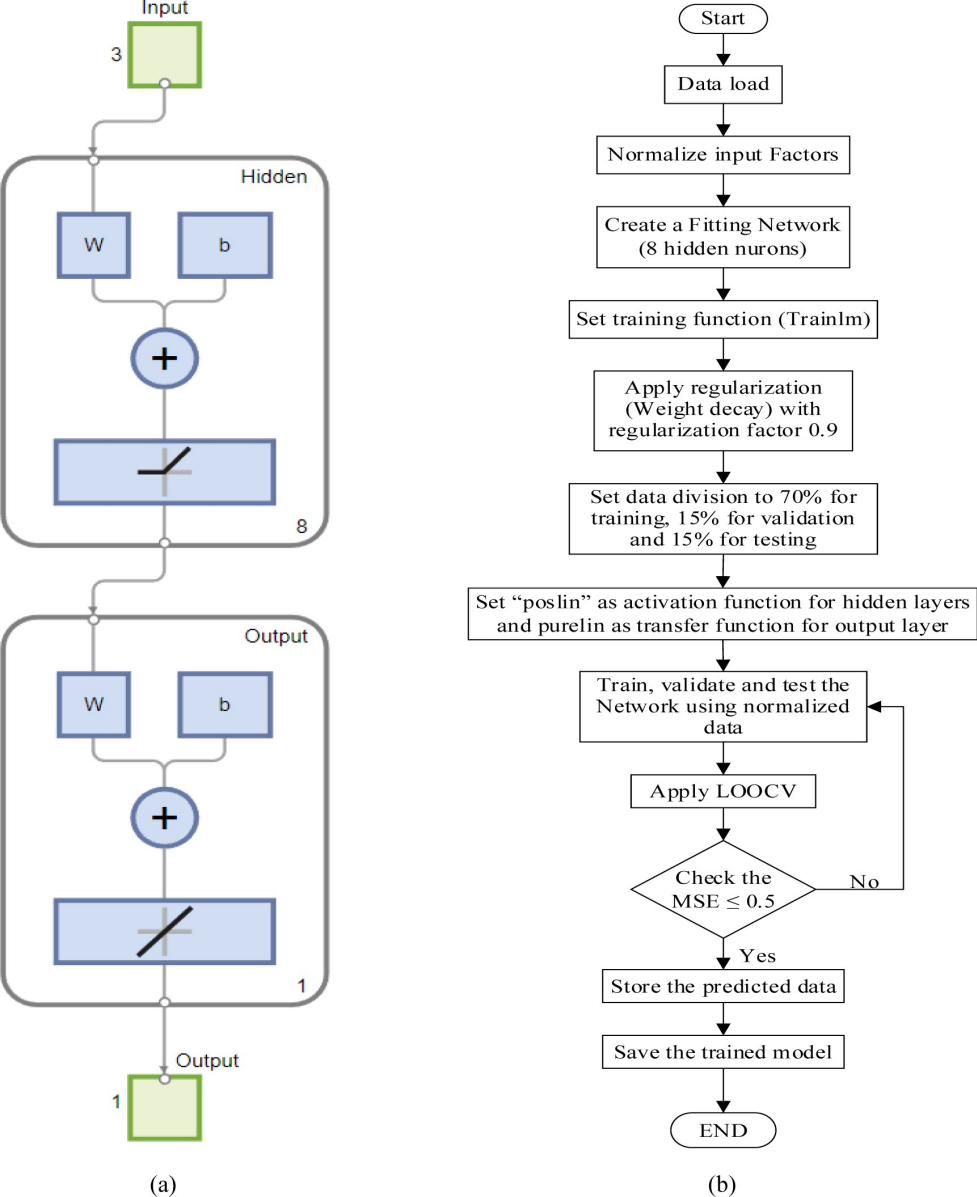
(a); ANN architecture, (b); Flowchart of ANN training process.

**Fig 9:**
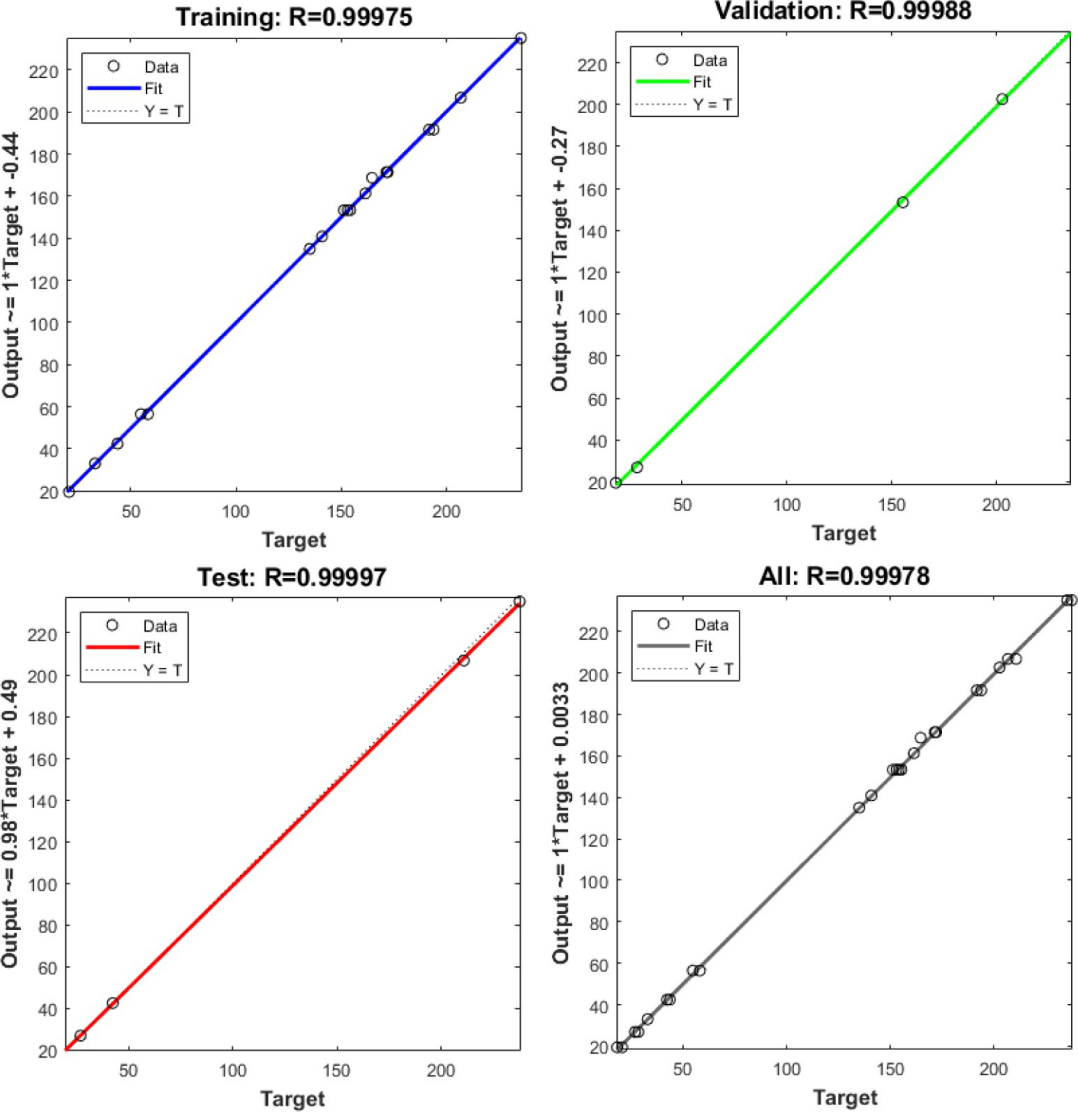
Scatter plots of ANN training, validation, testing and overall UTS dataset.

The backpropagation (BP) learning algorithm was utilized with a single hidden layer improved by a Levenberg-Marquardt numerical optimization technique and "poslin" activation function, which works well with small datasets. The transfer function for the layer’s output calculation from net input was set to linear (purelin). The purelin linear function is used for regression or numerical prediction problems where the output value is continuous, and the objective is to predict numerical values [65]. The ANN training performance was assessed by mean squared error (MSE) and coefficient of determination (R^2^). The optimal ANN architecture was found with eight neurons in the hidden layer, resulting in the 3-8-1 configuration shown in Fig 8(A). Fig 8(B) illustrates the flow of the ANN training process. The trained ANN model was saved when the lowest MSE (0.443) of the trained network performance was achieved, and the desired scatter plot regression line was obtained, as depicted in Fig 9. The scatter regression plot of ANN training revealed remarkable outcomes with high R^2^s values of 0.999 for the training, validation, testing set, and overall process, respectively. These values reflect training accuracy, robust generalizability, and consistency across diverse data subsets, indicating the model’s high prediction.

Integrating GA and RSM allows for more effective optimization of process parameters. GA is inspired by the principles of selection and evolution and operates by iteratively enhancing a population of candidate solutions through mutation, crossover, and selection until an optimal or near-optimal solution is attained [49]. RSM is a statistical modeling approach for analyzing and optimizing processes by fitting response surfaces to experimental data [66]. To integrate GA into the optimization process, the RSM model was used as a fitness function to evaluate the performance of different solutions. The RSM-GA approach aims to determine the input factor combination that results in the highest tensile strength.

### 2.4 Experimental tensile test

In order to analyze the effect of experimental process parameters on chip consolidation, the tensile test specimens were prepared according to the ASTM E8 M subsize. The tensile test was conducted using a Universal Testing Machine (Shimadzu EHF-EM0100K1-020-0A), chosen for its ability to perform precise tensile tests and ensure reliable and consistent data collection. The tensile test was conducted with an initial strain rate of 2.5 x 10^−3^ s^-1^ and pulled to failure at room temperature under 1 kg load.

## 3. Results and discussion

The experiments were conducted according to the run order outlined in section 2.1 Table 5, and the corresponding UTS results for each factor combination were collected. The curvature effect was found to be statistically significant upon ANOVA analysis of the 19 experiments’ results. This means the linear model may not accurately represent the relationship between independent and dependent variables. In other words, the response is not affected by any curved pattern in the independent factors. Therefore, ANOVA results suggested a further analysis of the higher order model. To facilitate this, six CCD experiments were added to the previously done factorial design for RSM analysis. Additionally, two center points were added to experimental designs to estimate the curvature effect better.

### 3.1 Ultimate tensile strength

**Fig 10:**
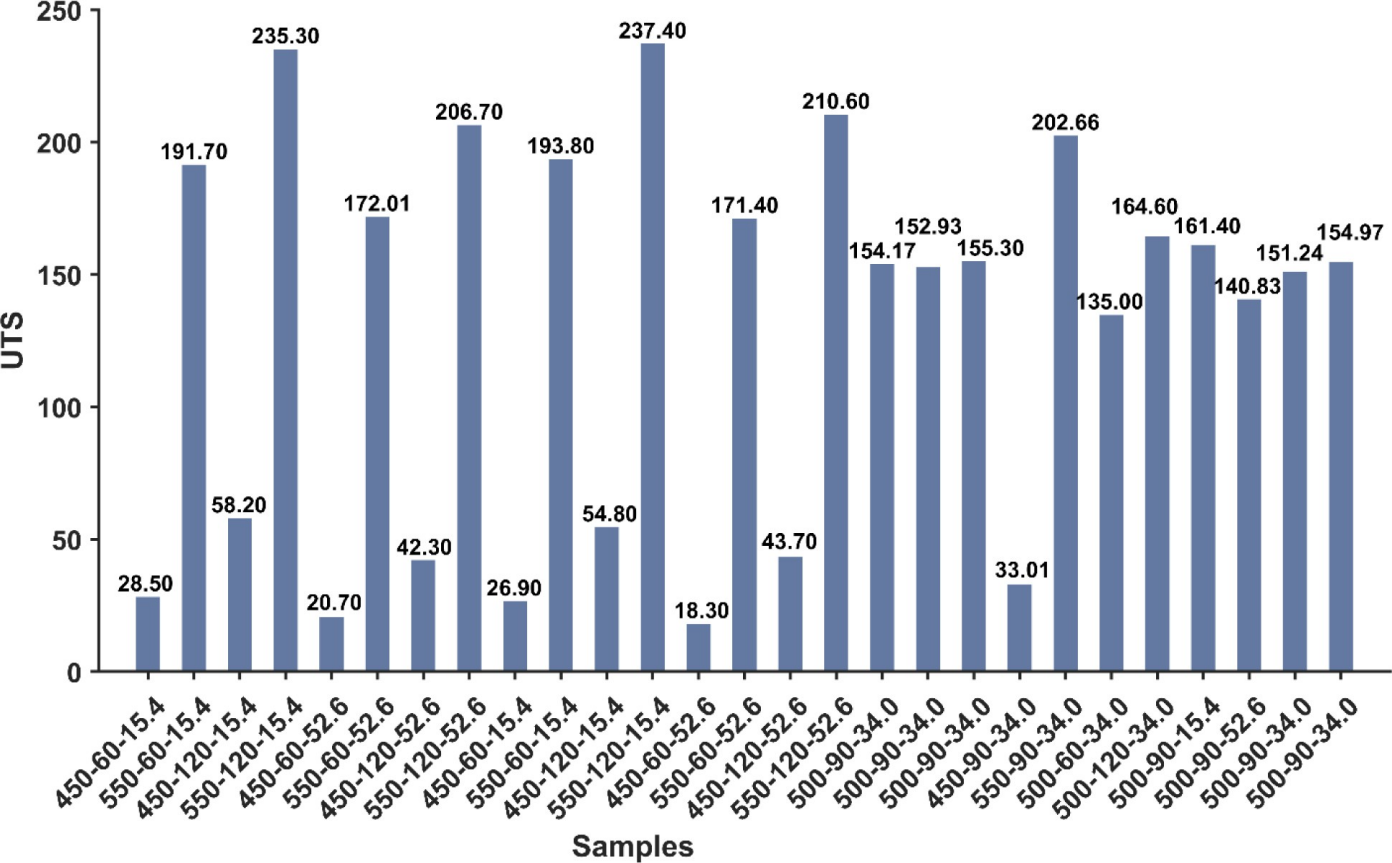
The UTS result based on different process parameters.

The UTS results of samples are plotted in a grouped bar chart, as shown in Fig 10. The x-axis denotes the input of process parameters, and the y-axis is the UTS (MPa). The graph illustrates the relationship between the combination of process parameters and UTS. In this study, the UTS was examined in the context of three process parameters: forging Temperature (Tp), holding time (HT), and the chip surface area (CSA). Deviations in UTS values are attributable to process parameter variations and their interactions. Significant outliers and variations in the UTS value in the bar heights allow for several insightful observations. The maximum UTS value of 237.4 MPa was obtained in a sample processed with 550°C, 120 min and 15.4 mm^2^ of CSA, while the lowest UTS value is linked to 450°C, 60 min and 52.6 mm^2^ of CSA. The highest temperature, at 550°C, was observed to exert the most influence on UTS results, regardless of the holding duration and chip size. At elevated temperatures, recrystallization was accelerated, resulting in smaller, more equiaxed grains with improved mechanical properties. The forging process deforms the material plastically, breaking the grains and forming smaller grains. As the forging temperature increases, the extent of plastic deformation increases, further reducing grain size [11, 67]. Additionally, the homogenization of the microstructure contributed to enhancing tensile strength.

Consequently, the rise in forging temperature correlates with increased UTS. It also improved material flow, minimized defects, and enhanced grain bonding. These arguments are supported by experimental investigations on the mechanical performance of aluminum chip-based recycled products [15, 16, 68].

However, prolonged forging to 120 minutes at high temperatures enhanced the UTS. There was an observable increase in the UTS of samples with longer HT than those with shorter ones. The grain size is induced by the holding time, in which a longer forging duration allows for increased grain growth [55]. However, when the chip’s surface area is small, the grains tend to proliferate excessively, resulting in brittle recycled material. As a result, the long duration of holding resulted in a further rise in UTS due to grain refining.

Another underlying parameter is the CSA. The findings showed that the larger surface area of the chip (corresponding to small chip size) is associated with lower UTS values. The UTS of large CSA-based recycled samples was lower than those recycled from small CSA because of the inverse relationship between the CSA and grain size. At elevated forging temperature and high deformation, smaller CSA exhibited finer grains and fewer grain boundaries, which act as barriers to dislocation movement and contribute to strengthening the material due to crystallographic changes [16].

Moreover, it can be attributed to oxide formation layers and microstructural variations. A larger CSA size tends to introduce more oxide amount, stress concentrators, and variations in the material’s microstructure, resulting in poor bonding. Researchers support and emphasize these justifications that the larger chip surface areas based on smaller chip size less than 2 mm in length negatively impact the strength of aluminum recycled parts [21, 55, 69]. The chips are significantly strengthened when exposed to high temperatures. It was observed that the samples produced from large and small CSA under high-temperature conditions demonstrated comparable strength. The heat increment enhanced the material’s plasticity, facilitating better consolidation. Additionally, promotes higher diffusion, which results in strong interfacial adhesion between the chips. Consequently, voids are reduced, and overall strength is improved [4–6].

In summary, findings showed that the forging temperature had the most significant impact on UTS, followed by holding time and chip surface area. Optimizing these process parameters can further enhance the material’s strength by considering other influencing factors and conducting further research to validate these findings in practical applications.

### 3.2 Analysis of Variance (ANOVA) of UTS

Analyzing and identifying the variations in results can provide valuable insights for process optimization and control. The final ANOVA for tensile strength is presented in Table 6. The backward elimination method was employed with a significant threshold to ensure the exclusion of nuisance (non-significant factors) while retaining the primary factor in the model. This method reduces the model’s complexity and enhances its interpretability [48]. By eliminating non-significant factors, the model becomes more efficient and concentrates on the factors significantly affecting the response.

**Table 6:** ANOVA result of UTS.

Source	DF	Adj SS.	Adj MS	F-Value	P-Value	
Model	8	137435	17179	4968.30	0.000	Significant
Linear	3	130487	43496	12579.07	0.000	
Tp	1	124195	124195	35917.39	0.000	
HT	1	4844	4844	1400.96	0.000	
CSA	1	1448	1448	418.85	0.000	
Square	1	4710	4710	1362.02	0.000	
Tp*Tp	1	4710	4710	1362.02	0.000	
2-Way Interaction	3	418	139	40.30	0.000	
Tp*HT	1	199	199	57.68	0.000	
Tp*CSA	1	183	183	52.88	0.000	
HT*CSA	1	36	36	10.33	0.005	
Error	18	62	3			
Lack-of-Fit	7	29	4	1.40	0.296	Insignificant
Pure Error	11	33	3			
Total	26	1375				
Standard deviation = 5.00, *R*^2^ = 99.9%, *R*^2^ adjusted = 99.9%, *R*^2^ predicted = 99.8%						

DF is the degree of freedom, Adj SS is the adjacent sum of squares, Adj MS is the adjacent mean squares, and the *p-value* is the significance level.

In ANOVA analysis, the probability value (p-value) is vital in assessing the significance of each factor in the model. The p-value determines whether or not that factor has a statistically significant influence on the output response by testing the null hypothesis for each term when the coefficient has no effect [57]. The statistical significance level of the p-value (typically <0.05) indicates that the null hypothesis can be rejected, as the coefficient is deemed equal to zero. The p-values of all terms in the ANOVA results (Table 6) were less than 0.05, indicating that the UTS model is statistically significant. The model effectively represents the relationship between input and output variables, as indicated by the highly significant model fit (p < 0.05). The Lack-of-fit refers to the discrepancy between the model and the relationship between dependent and independent variables. In this case, the insignificant lack-of-fit value in the ANOVA result indicates that the model is appropriate and accurately describes the functional relationship between input factors and response [57]. The coefficient of determination (R^2^) denotes the proportion of variation in the response variable that the model can explain. The R^2^ value of 99.9% in the given model summary indicates that the model accounts for a high proportion of the variability in the response variable. The high value shows the ability of the model to explain the observed variation in the output responses firmly.

**Fig 11:**
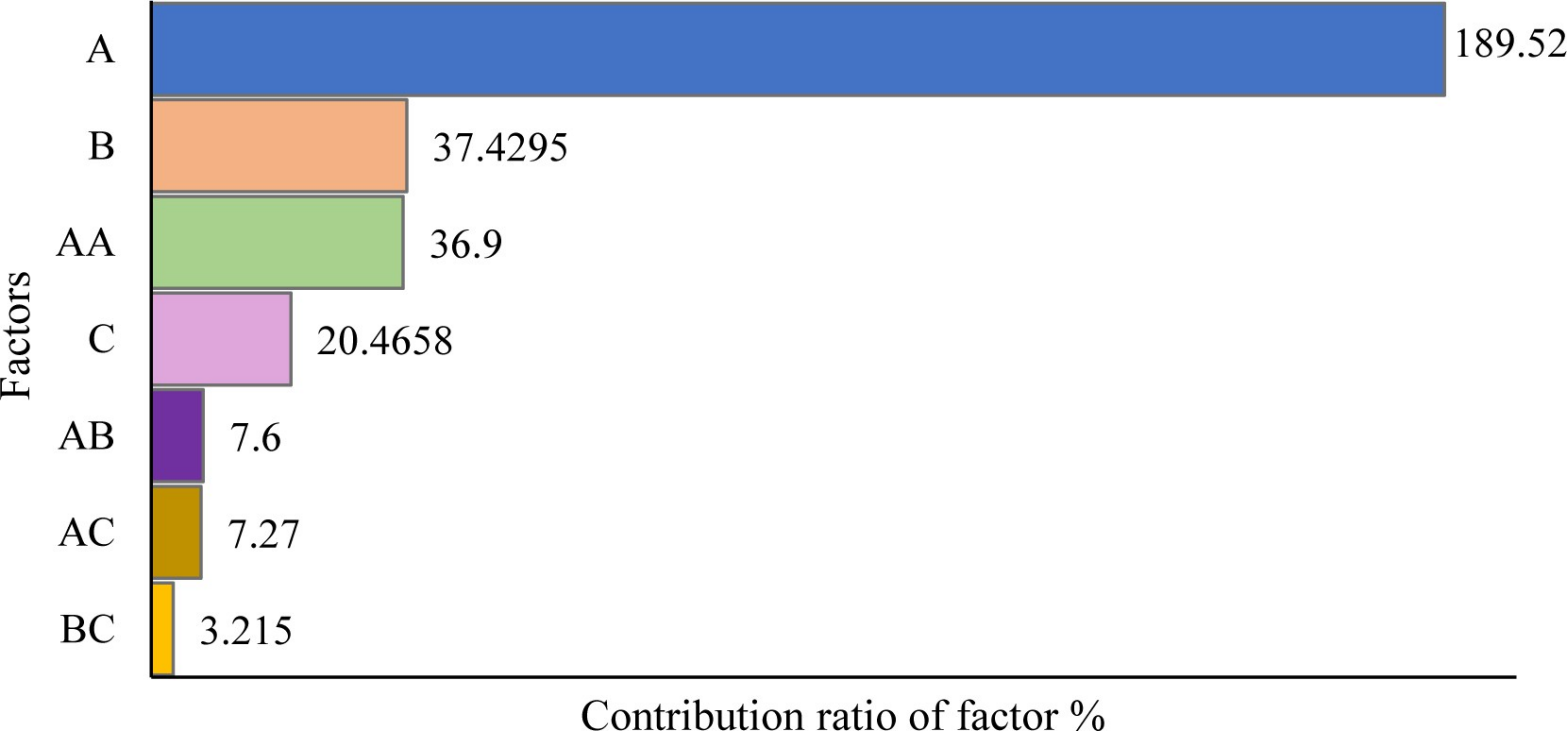
Pareto chart of factor contribution effect ratio on UTS.

Fig 11 displays the ANOVA Pareto chart of the standardized effects contribution ratio of the input factors and their interaction. The operating temperature, holding time, and chip surface area are denoted by A, B and C, respectively. The length of each bar indicates the effect ratio of the factor. As observed, temperature (A) had the highest contribution effect on the UTS of the recycled part, as indicated by the large ratio of 189.52, followed by holding time (B) and the interaction between temperatures (AA). Moreover, the chip surface area (C) exhibited a significant effect, albeit to a lesser extent than A and B. The AB and AC interactions also showed a relatively minor effect, while the BC interaction had no significant effect on the UTS. Higher forging temperature and time enhance the diffusion rate in the chip, improving adhesion and uniformity [70]. Additionally, a larger chip promotes higher contact points, increasing adhesion strength [71, 72].

**Fig 12:**
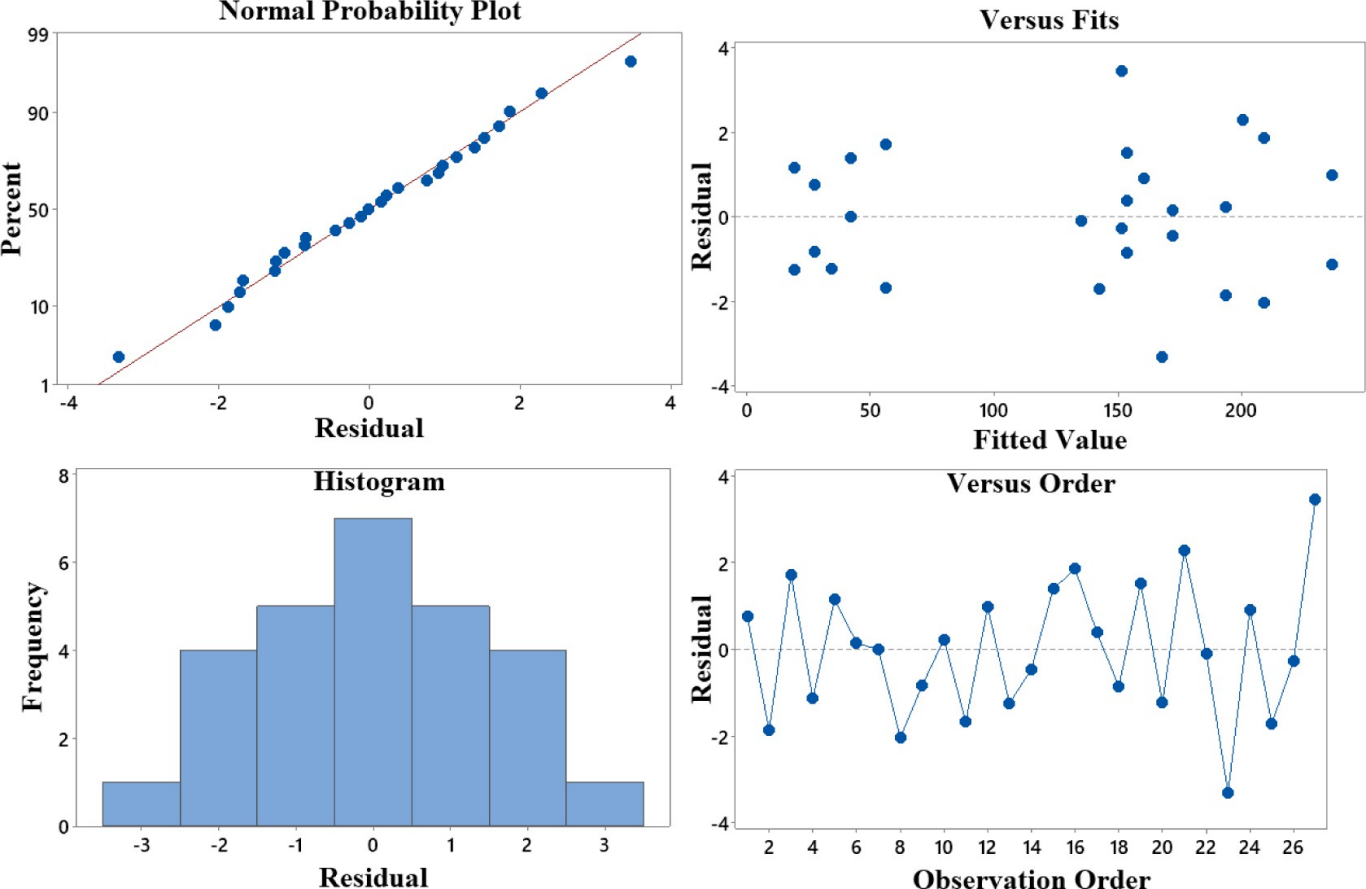
Residual plot for UTS.

The residual plot in Fig 12 shows that most scatter points fell on the line, suggesting that the regression model fits the data well. There is a slight trend of the residuals increasing as the fitted values increase. This indicates that the regression model underestimated the UTS values for higher-fitted values. However, the residuals are still randomly scattered around the line, suggesting that the regression model fits the data well.

**Fig 13:**
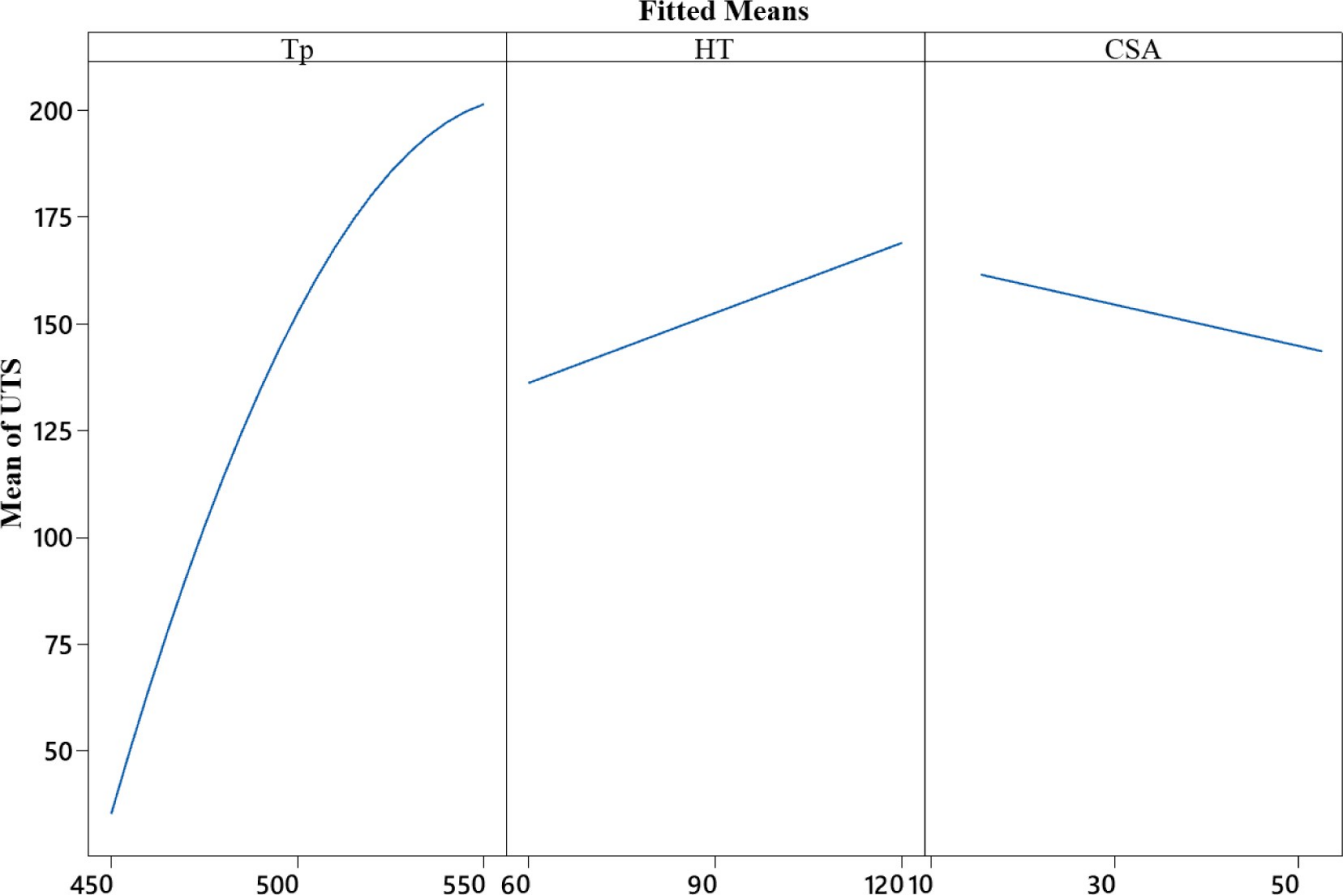
Main effect plot for UTS.

The ANOVA main effect plot in Fig 13 illustrates the positive correlation between the UTS with forging Temperature (Tp) and Holding time (HT). However, the relationship between UTS and the size chip’s SA is inverse; the average UTS score was higher with a smaller SA. This can be attributed to reduced oxide formation on smaller SA, as the higher formation of the aluminum oxide layer acts as weak points, causing poor bonding and lower UTS.

### 3.3 Modeling and optimization result

Modeling and optimizing the processing parameters can result in the desired material strength. The ultimate tensile strength was modeled and optimized using the ANN and RSM-GA approach.

#### 3.3.1 RSM vs. ANN models in UTS prediction

The RSM empirical model was developed to describe the relationship between the output response and the input factors in the model. The model was derived based on experimental data analysis using regression techniques. The model can predict the UTS for new values of the input variables (Tp, HT, and CSA). The final regression model is expressed in (3).


$$UTS\;(MPa)=‐4095.0+15.253\;(Tp)‐0.539\;(HT)+1.576\;(CSA)‐0.013680\;{\left(Tp\right)}^{2}+0.002354\;(Tp)\;(HT)‐0.003635\;(Tp)\;(CSA)‐0.0026878\;(HT)\;(CSA)$$ (3)

The coefficients (including 15.253, -0.539, 1.576, -0.01368, 0.002354, -0.003635 and -0.002678) represent the estimated effects of the variables on UTS.

The developed RSM empirical model accurately predicted the UTS of chip-based forged parts under various untested conditions and provided valuable insight into tensile strength prediction. The prediction accuracy was assessed using the coefficient of determination (R^2^), mean square error (MSE), root mean square error (RMSE) and mean absolute error (MAE) metrics shown in Eqs 4–8.


$$R^{2}=\frac{\sum_{i=1}^{n}({y_{ai}-y_{pi})}^{2}}{\sum_{i=1}^{n}({y_{pi}-y_{a.mn})}^{2}}$$ (4)

$$R^{2}‐adjusted=\left[(1-R^{2})\times \frac{n-1}{n-k-1}\right]$$ (5)

$$MSE=\frac{1}{n}\sum_{i=1}^{n}{{(y}_{pi}-y_{ai})}^{2}$$ (6)

$$RMSE=\sqrt{\frac{1}{n}\sum_{i=1}^{n}{{(y}_{pi}-y_{ai})}^{2}}$$ (7)

$$MAE=\frac{1}{n}\sum_{i=1}^{n}\left|\left(y_{ai}-y_{pi}\right)\right|$$ (8)

Where and represent the actual and predicted values, respectively. The denotes the mean value of the observed response, k and n are the number of input factors and observed data points, respectively.

**Fig 14:**
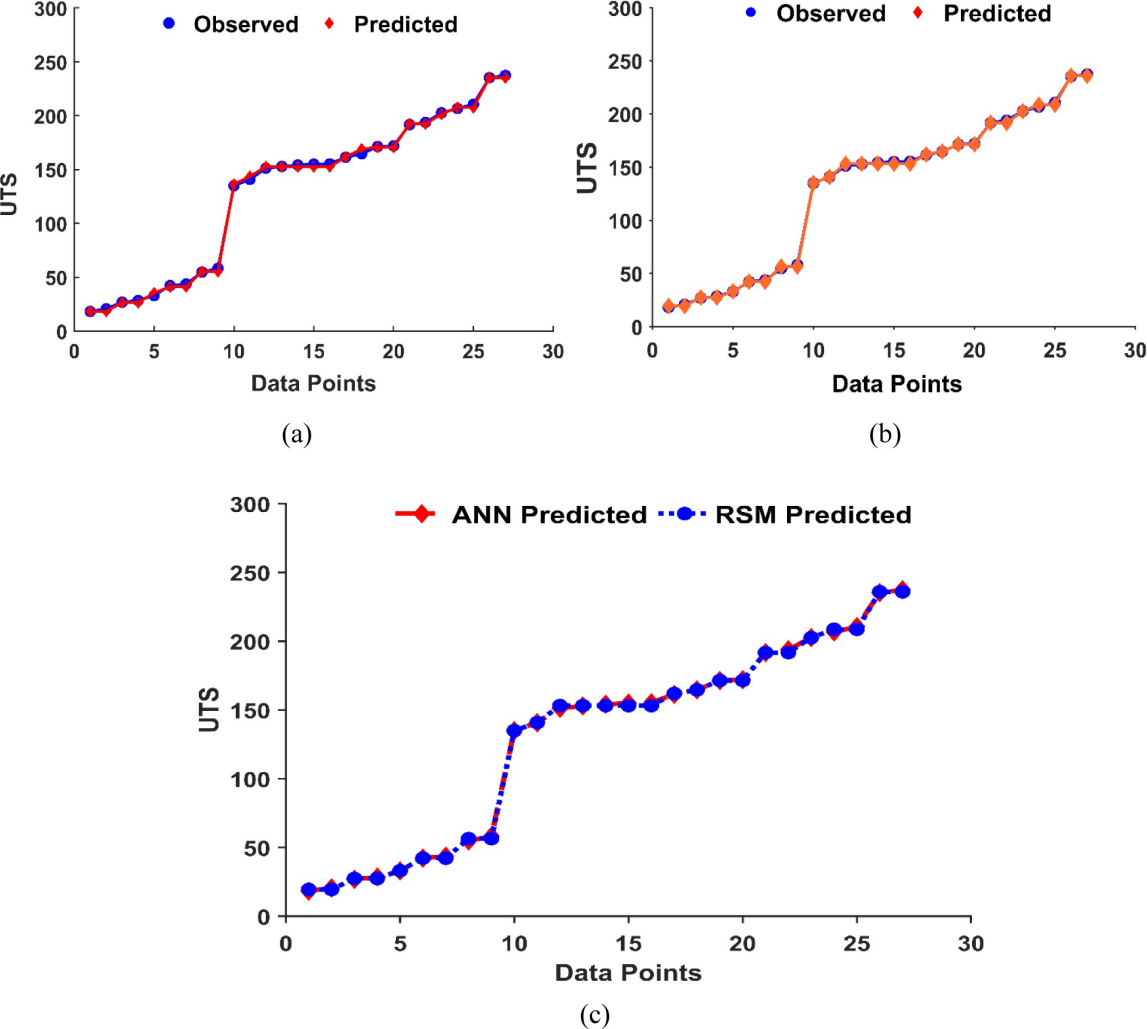
(a); Observed vs. predicted UTS by RSM, (b); by ANN model and (c); RSM vs ANN model data training performance.

The RSM prediction’s accuracy evaluation yielded promising results, showcasing an MSE of 3.6%, RMSE of 1.89%, MAE of 1.56%, and R^2^ value of 99.9%, as outlined in Table 7. The small value of metrics result signified a good performance of the predictive model in estimating UTS values, although further comparisons with other models are recommended to validate its superiority. Overall, the results highlighted the model’s accuracy in predicting the UTS response compared to the observed data, as plotted in Fig 14(A).

**Table 7:** Evaluation metrics for the accuracy of data predicted by the RSM and ANN models.

Metrics	RSM Model	ANN Model
MSE (%)	3.595	1.476
RMSE (%)	1.896	1.214
MAE (%)	1.562	0.944
R^2^ (%)	99.9	0.999

**Fig 15:**
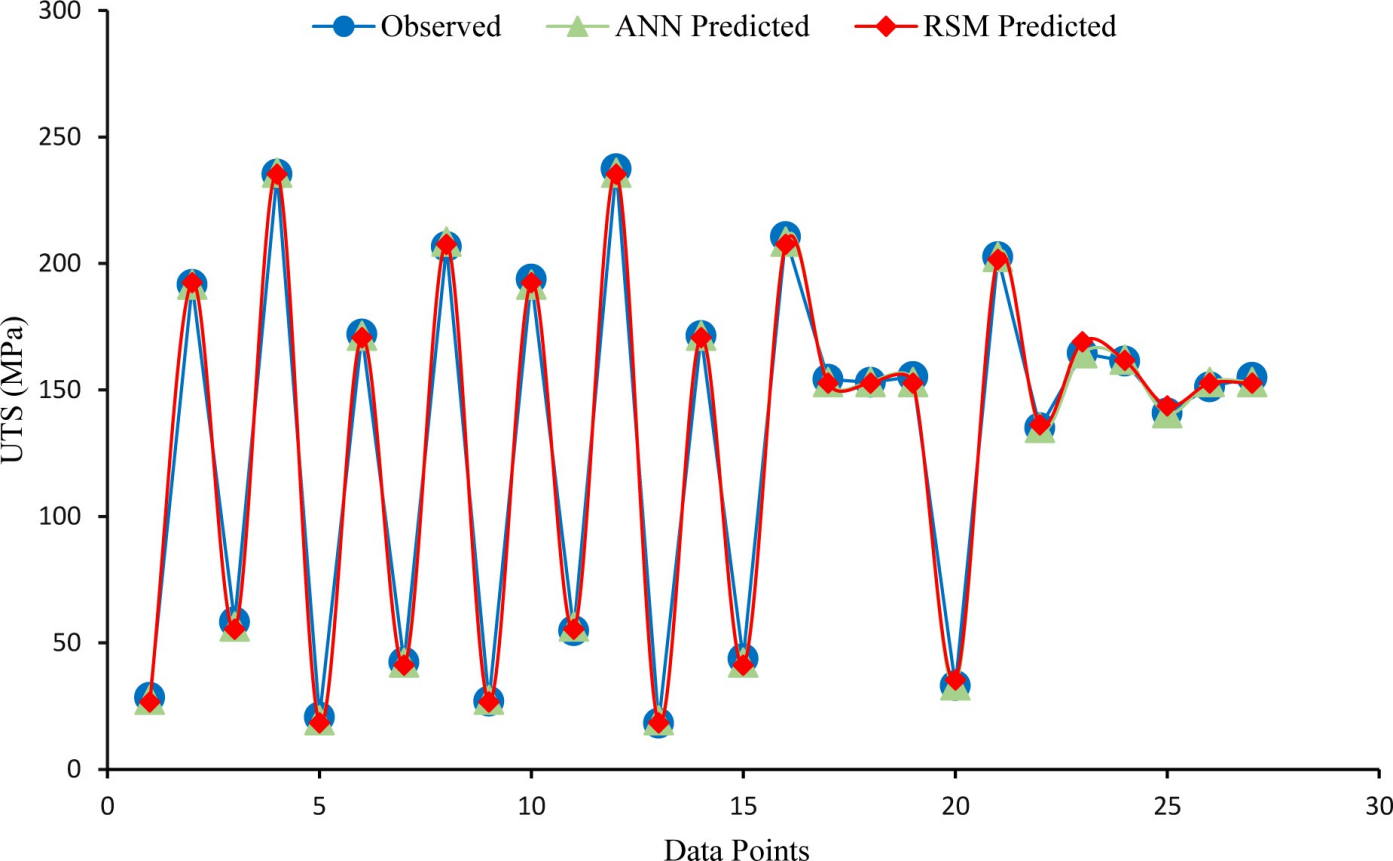
The observed UTS versus predicted by RSM and ANN model.

The ANN model was used to predict UTS values based on a combination of input factors. The accuracy of predicted data was evaluated by MSE, RMSE, MAE, and R^2^ metrics, as shown in Table 7. These metrics provide different perspectives on the accuracy of the prediction. The evaluation metrics between actual and predicted UTS revealed that ANN prediction is more accurate than RSM. While both models exhibited slightly close prediction performances, the ANN outperformed RSM. The predicted values demonstrated that the trained ANN model was able to simulate the UTS results of recycled samples based on various combinations of Tp, HT and CSA process parameters, as given in Fig 14(B). The predicted UTS values of RSM and ANN models are listed in Table 8, along with input factors, observed UTS and the percentage error between predicted and actual values. The RSM and ANN predicted data were plotted versus the observed UTS, as shown in Fig 15.

**Table 8:** Experimental design matrix with actual and predicted UTS values.

Run Order	Input Factors			Experiment UTS	Predicted UTS		% error ×100	
	Tp (°C)	HT (min)	CSA mm/mm^2^		RSM	ANN	RSM	ANN
1	450	60	15.4	28.50	26.6050	27.4730	6.649	3.604
2	550	60	15.4	191.7	192.434	191.709	-0.38	-0.005
3	450	120	15.4	58.20	55.3430	56.4720	4.91	2.969
4	550	120	15.4	235.3	235.294	235.949	0	-0.276
5	450	60	52.6	20.70	18.4150	19.4940	11.04	5.826
6	550	60	52.6	172.01	170.721	171.435	0.75	0.334
7	450	120	52.6	42.30	41.1750	42.2730	2.66	0.064
8	550	120	52.6	206.7	207.604	208.660	-0.44	-0.948
9	450	60	15.4	26.90	26.6050	27.4730	1.1	-2.13
10	550	60	15.4	193.8	192.434	191.709	0.7	1.079
11	450	120	15.4	54.80	55.3430	56.4720	-0.99	-3.051
12	550	120	15.4	237.4	235.294	235.949	0.89	0.611
13	450	60	52.6	18.30	18.4150	19.4940	-0.63	-6.525
14	550	60	52.6	171.4	170.721	171.435	0.4	-0.02
15	450	120	52.6	43.70	41.1750	42.2730	5.78	3.265
16	550	120	52.6	210.6	207.604	208.660	1.42	0.921
17	500	90	34.0	154.2	152.650	153.165	1.01	0.671
18	500	90	34.0	152.93	152.650	153.165	0.18	-0.154
19	500	90	34.0	155.3	152.650	153.165	1.71	1.375
20	450	90	34.0	33.01	35.3840	33.1590	-7.19	-0.451
21	550	90	34.0	202.66	201.513	202.620	0.57	0.02
22	500	60	34.0	135.0	136.245	134.982	-0.92	0.013
23	500	120	34.0	164.6	169.055	164.595	-2.71	0.003
24	500	90	15.4	161.4	161.620	161.966	-0.14	-0.351
25	500	90	52.6	140.83	143.680	140.782	-2.02	0.034
26	500	90	34.0	151.24	152.650	153.165	-0.93	-1.273
27	500	90	34.0	154.97	152.650	153.165	1.5	1.165

#### 3.3.2 RSM vs. GA optimization process

**Fig 16:**
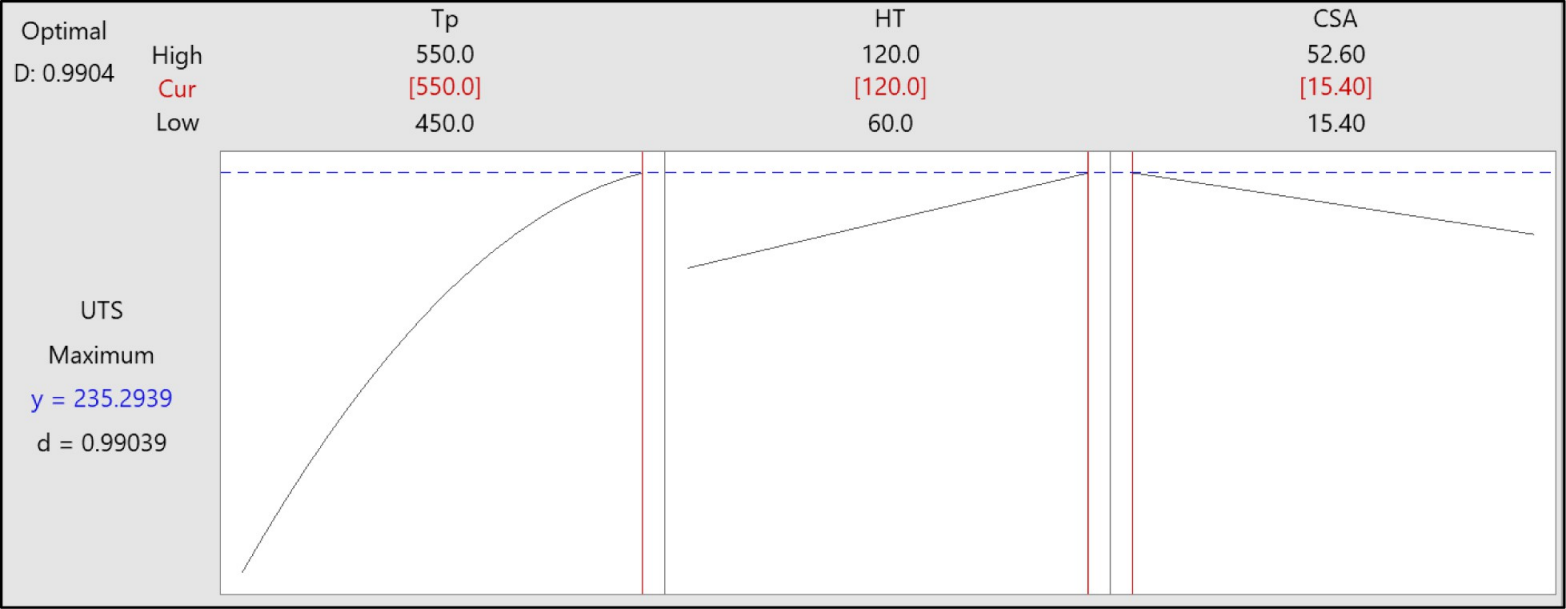
RSM optimization plot for UTS.

RSM and GA optimization were executed to determine the optimum processing parameters and maximize UTS response. RSM-GA can provide advantages over performing either method alone, mainly when the optimization process is intricate and the parameter space has a high dimension. The RSM analyzed the input factors to acquire the optimal values that result in maximum UTS. Table 9 and Fig 16 depict the optimal parameters and response derived from the RSM optimization process. The solution suggested 550°C, 120 min, and 15.4 mm^2^ as the optimal parameters for attaining UTS values of 235.3 MPa. The optimization result corresponds to the experimentation findings. The composite desirability evaluates the solution’s quality from 0 to 1, with 1 being the optimal solution [57]. The desirability of 0.99, in this case, indicates good solution performance across the considered criteria.

**Table 9:** RSM optimal solution.

Tp	HT	CSA	UTS Fit	Composite Desirability
550	120	15.4	235.3	0.99

**Fig 17:**
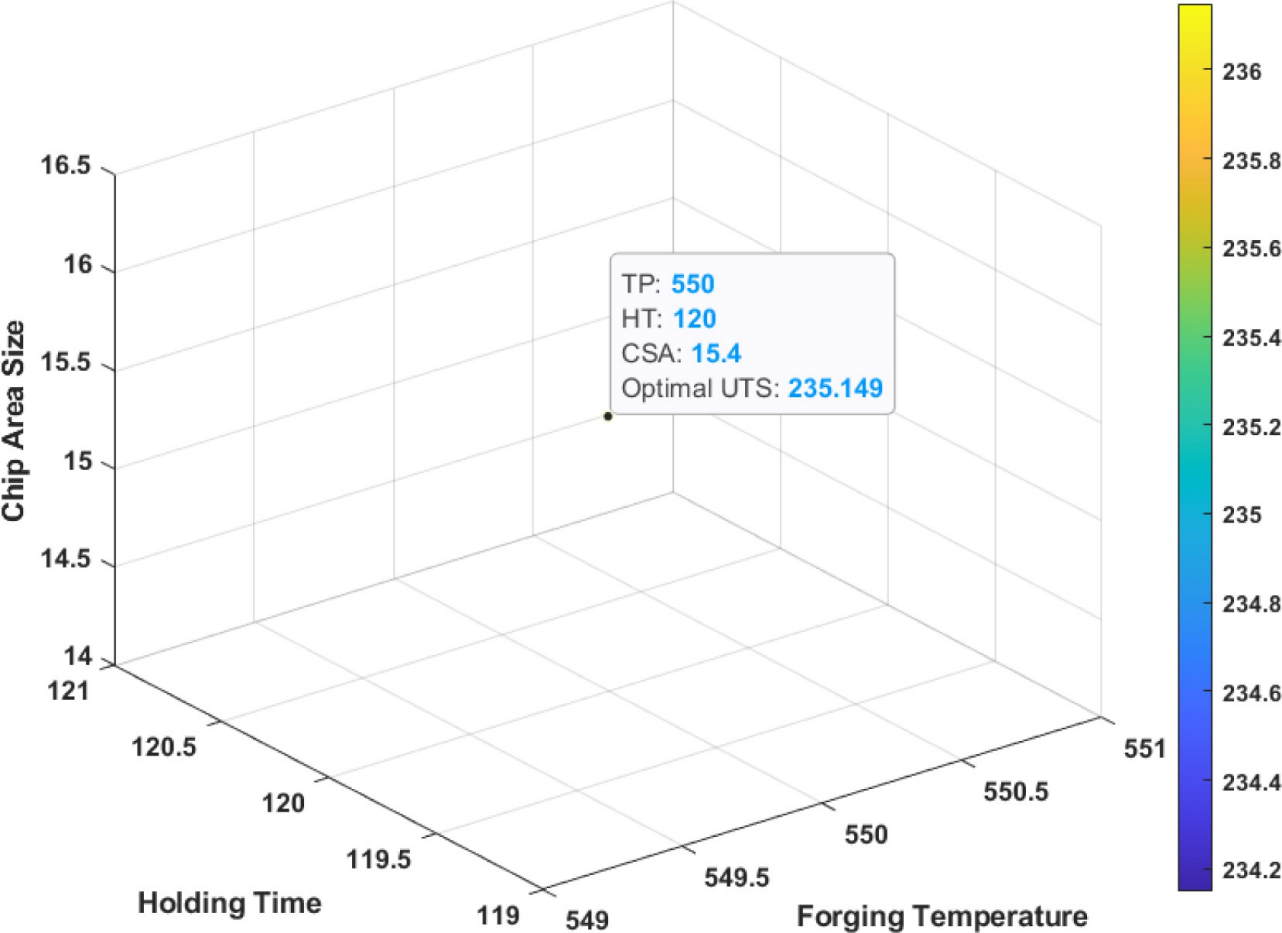
GA-RSM optimization plot for UTS.

In the GA optimization process, the maximum number of generations and population size were set to 100 and Function Tolerance to 10^−6^. The number of variables to optimize was 3; Tp, HT, and CSA with lower and upper limits. The optimization was terminated when the maximum number of generations was reached. The GA process iteratively evaluated different combinations of input factors and calculated the corresponding tensile strength by employing the ANN and RSM models. The ideal condition was 550°C, 120 min, and 15.4 mm^2^, while the optimal UTS was 235.15 MPa, as depicted in the GA-RSM optimization plot in Fig 17 and Table 10. The GA-ANN optimization result was identical to the GA-RSM result, except the optimal UTS was 236.2. The optimal process variables and response were consistent with the experiment result. Remarkably, both approaches generated identical optimal input and output values, signifying reliable and consistent convergence toward the best solution. This finding supports the idea that GA can yield accurate results. The optimization process is considered robust as it consistently converges to the identical optimal solution, validating the efficacy of the methodology.

**Table 10:** The optimal conditions of process parameters and response by RSM and GA-ANN optimization processes.

Method	Optimal parameters			Optimal response
	TP	HT	CSA	UTS
RSM	550	120	15.4	235.30
GA-RSM	550	120	15.4	235.15
GA-ANN	550	120	15.4	236.20

### 3.4 Confirmation test and validation

**Fig 18:**
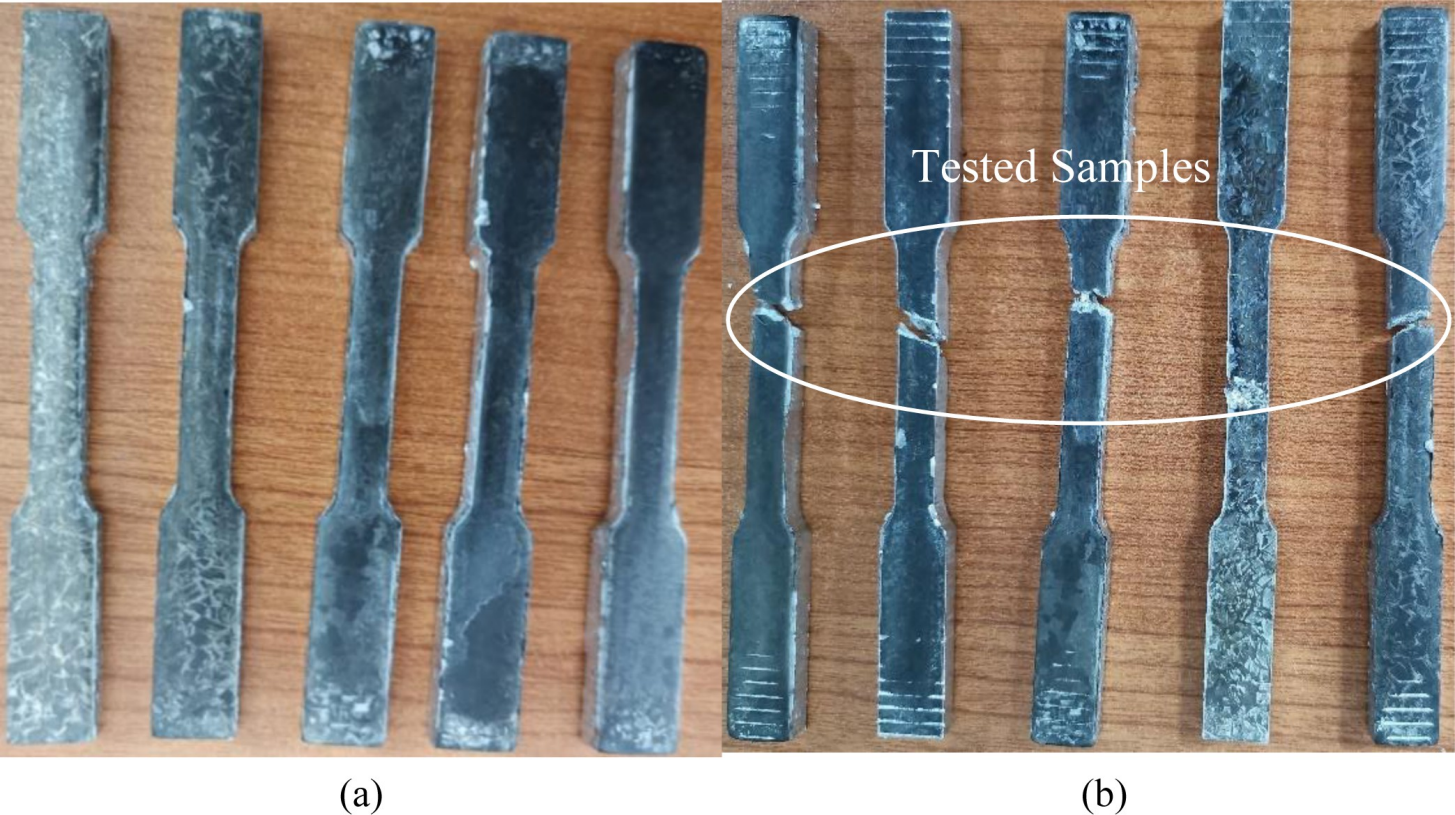
The confirmatory experiment sample (a) before and (b) after testing.

The optimum settings outlined in Table 10 were validated experimentally. Five samples were processed based on optimal parameters and tested for tensile strength, as presented in Fig 18. The obtained UTS results of the five samples (Table 11) corresponded to the RSM, GA-RSM and GA-ANN optimum UTS values. The calculated error % between the averaged UTS value of five experimental samples and the optimized values was found to be less than 2% for the ANN model, as listed in Table 11. This result confirmed the reproducibility of the experimental data. Thus, the predicted UTS agreed well with the observed UTS.

**Table 11:** The percentage error between confirmatory samples and RSM with GA result.

Exp. No.	UTS values			Error %×100	
	Experimental	RSM Model	ANN Model	RSM	ANN
S1	238.07	235.3	236.2	1.164	1.177
S2	234.16			0.487	0.484
S3	230.76			1.967	1.929
S4	228.28			3.075	2.983
S5	237.80			1.051	1.062
Average	233.814			1.55%	1.63%

The percentage error between confirmatory experiments and the empirical result was calculated using the formula in Eq (9) as follows:

$$Error\;\%=\frac{\left|E-P\right|}{E}x\;100$$ (9)

Where and denote experimental and predicted values, respectively.

## 4. Conclusion

This paper investigated the strength of AA6061 chip-based forged parts prepared by the solid-state recycling method. The effect of forging temperature, holding time, and chip’s SA factors were investigated. The high forging temperature and prolonged holding time enhanced tensile strength by promoting chip welding. Moreover, findings exhibited that the higher chip SA is associated with inferior UTS values, possibly due to the presence of oxide layers. Based on the findings, the main conclusion can be summarized as follows:

- The highest UTS of 237.4 MPa was recorded based on 550°C and 120 min, and 15.4 mm^2^ of Tp, HT and CSA, respectively. In contrast, the lowest UTS value was achieved at 450°C, 60 min and 54.6 mm^2^.
- ANOVA analysis revealed that both factors had a statistically significant influence on the UTS response. However, the forging temperature and its interaction had higher contribution ratios.
- The RSM, GA-RSM and GA-ANN optimization processes suggested 550°C, 120 min, and 15.4 mm^2^ are the optimal processing parameters. Notably, the ideal UTS response was nearly identical across all optimization methods.
- The accuracy of optimized processing parameters was experimentally confirmed. The error % between confirmatory experiment samples and the optimized values was less than 1%.
- The deviation between actual and predicted UTS values by RSM and ANN models was minimal, with an average error of less than 2%.
- The statistical metrics evaluation of predicted results indicated that the ANN model was more accurate in simulating UTS values across different combinations of process parameters.

A study on the chip size and surface area over the resulting recycled strength provides a valuable guideline for industry practitioners implementing the HPF process to estimate the strength of chip-based recycled products. They can control the processing parameters, such as forging temperature and time, to produce the desired strength of recycled products by selecting optimal chip sizes using the developed model. Future investigations could consider expanding this method to diverse alloys and manufacturing techniques, thereby achieving a comprehensive comprehension and practicality across multiple sectors in the metalworking field. Further future work is warranted to model the mechanism of aluminum chip bonding in the HPF recycling process. This can contribute to enhancing the strength of chip welding and overall quality. This research contributes to promoting sustainable manufacturing and highlights the potential of integrating the RSM statistical method with the predictive power of machine learning techniques to optimize complex manufacturing processes.

## Supporting information

S1 File (DOCX)

S1 Data (DOCX)
