# Correction: Optimizing strength of directly recycled aluminum chip-based parts through a hybrid RSM-GA-ANN approach in sustainable hot forging

**DOI:** 10.1371/journal.pone.0362612

**Published:** 2026-11-11

**Authors:** Yahya M. Altharan, Shazarel Shamsudin, Mohd Amri Lajis, Sami Al-Alimi, Nur Kamilah Yusuf, Nayef Abdulwahab Mohammed Alduais, Atef M. Ghaleb, Wenbin Zhou

In the Abstract section, there is an error in the ninth sentence. The correct sentence is: The proposed optimization process suggested 550°C, 120 minutes, and 15.4 mm^2^/mm^3^ as the optimal condition yielding the maximum UTS.

In Table 11, the ANN percentage-error values were incorrect. Please see the correct Table 11 below.

**Table 11:** The percentage error between confirmatory samples and RSM with GA result.

Exp. No.	UTS value (MPa)			Error (%) $=\;\frac{|E-P|}{E}\;$×100	
	Experimental	RSM Model	ANN Model	RSM	ANN
S1	238.07	235.3	236.2	1.164	0.785
S2	234.16			0.487	0.871
S3	230.76			1.967	2.357
S4	228.28			3.075	3.469
S5	237.80			1.051	0.673
Average	233.814			1.55%	1.63%
