# Supplementary material for: Optimizing strength of directly recycled aluminum chip-based parts through a hybrid RSM-GA-ANN approach in sustainable hot forging
Source: PLoS One. 2024 Mar 14;19(3):e0300504. doi: 10.1371/journal.pone.0300504 (PMC10939231; doi:10.1371/journal.pone.0300504)
Supplement: S1 File — (DOCX) [file pone.0300504.s001.docx]

[Prof. Dr. Emily Chenette](https://ijtech.eng.ui.ac.id/people)

Editor-in-Chief

PLOS ONE

[February 2, 2024]

PONE-D-23-37345R2

Dear Sir/Madam,

I hope you're doing well. Regarding the accepted manuscript titled " Optimizing Strength of Directly Recycled Aluminum Chip-Based Parts through a Hybrid RSM-GA-ANN Approach in Sustainable Hot Forging," I would like to request that only the following acknowledgment and finding statement be included in the final version:

**Acknowledgment**

"(Communication of this research is made possible through monetary assistance by Universiti Tun Hussein Onn Malaysia and the UTHM Publisher’s Office via Publication Fund E15216. The authors would also like to express the most profound appreciation for supplementary provisions provided by Sustainable Manufacturing and Recycling Technology, Advanced Manufacturing and Materials Center (SMART-AMMC), Universiti Tun Hussein Onn Malaysia.)"

"The authors extend their appreciation to Alfaisal University, Riyadh, Saudi Arabia."

**Funding Statement**

This research was supported by Universiti Tun Hussein Onn Malaysia (UTHM) through Tier 1
(Q390).

After careful consideration, I kindly request that the following funding statement and acknowledgment section not be published or included in the final version of the manuscript. Please remove it.

“The authors extend their appreciation to King Saud University for funding this work through Researchers Supporting Project number (RSPD2023R711), King Saud University, Riyadh, Saudi Arabia.) This research was funded by King Saud University through Researchers Supporting Project number (RSPD2023R711).”
