# Supplementary material for: Optimizing strength of directly recycled aluminum chip-based parts through a hybrid RSM-GA-ANN approach in sustainable hot forging
Source: PLoS One. 2024 Mar 14;19(3):e0300504. doi: 10.1371/journal.pone.0300504 (PMC10939231; doi:10.1371/journal.pone.0300504)
Supplement: S1 Data — (DOCX) [file pone.0300504.s002.docx]

Experimental design runs with UTS Test Results

| Run Order | Center Point | Input Factors | | |  | Response | Design Runs |
| --- | --- | --- | --- | --- | --- | --- | --- |
|  |  | Temp. (Tp) °C | Holding Time (HT) min | Chip Surface Area (CSA) mm^2^ |  | UTS |  |
| 1 | 1 | 450 | 60 | 15.4 |  | 28.500 | Full Factorial Design with 2 Replications and 3 Center Points |
| 2 | 1 | 550 | 60 | 15.4 |  | 191.70 |  |
| 3 | 1 | 450 | 120 | 15.4 |  | 58.200 |  |
| 4 | 1 | 550 | 120 | 15.4 |  | 235.30 |  |
| 5 | 1 | 450 | 60 | 52.6 |  | 20.700 |  |
| 6 | 1 | 550 | 60 | 52.6 |  | 172.01 |  |
| 7 | 1 | 450 | 120 | 52.6 |  | 42.300 |  |
| 8 | 1 | 550 | 120 | 52.6 |  | 206.70 |  |
| 9 | 1 | 450 | 60 | 15.4 |  | 26.900 |  |
| 10 | 1 | 550 | 60 | 15.4 |  | 193.80 |  |
| 11 | 1 | 450 | 120 | 15.4 |  | 54.800 |  |
| 12 | 1 | 550 | 120 | 15.4 |  | 237.40 |  |
| 13 | 1 | 450 | 60 | 52.6 |  | 18.300 |  |
| 14 | 1 | 550 | 60 | 52.6 |  | 171.40 |  |
| 15 | 1 | 450 | 120 | 52.6 |  | 43.700 |  |
| 16 | 1 | 550 | 120 | 52.6 |  | 210.60 |  |
| 17 | 0 | 500 | 90 | 34.0 |  | 154.20  Center Points |  |
| 18 | 0 | 500 | 90 | 34.0 |  | 152.93 |  |
| 19 | 0 | 500 | 90 | 34.0 |  | 155.30 |  |
| 20 | -1 | 450 | 90 | 34.0 |  | 33.010 | Additional Axial Points-RSM Runs with 2 Center Points |
| 21 | -1 | 550 | 90 | 34.0 |  | 202.66 |  |
| 22 | -1 | 500 | 60 | 34.0 |  | 135.00 |  |
| 23 | -1 | 500 | 120 | 34.0 |  | 164.60 |  |
| 24 | -1 | 500 | 90 | 15.4 |  | 161.40 |  |
| 25 | -1 | 500 | 90 | 52.6 |  | 140.83 |  |
| 26 | 0 | 500 | 90 | 34.0 |  | 151.24 |  |
| 27 | 0 | 500 | 90 | 34.0 |  | 154.97 |  |

Experimental design matrix with actual and predicted UTS values

| **Run Order** | **Input Factors** | | |  | **Experiment UTS** |  | **Predicted UTS** | |  | | **% error**$\boldsymbol{=}\frac{\boldsymbol{E-P}}{\boldsymbol{E}}$ **×100** | |
| --- | --- | --- | --- | --- | --- | --- | --- | --- | --- | --- | --- | --- |
|  | **Tp (°C)** | **HT (min)** | **CSA mm/mm^2^** |  |  |  | **RSM** | **ANN** | |  | **RSM** | **ANN** |
| 1 | 450 | 60 | 15.4 |  | 28.50 |  | 26.6050 | 27.4730 | |  | 6.649 | 3.604 |
| 2 | 550 | 60 | 15.4 |  | 191.7 |  | 192.434 | 191.709 | |  | -0.38 | -0.005 |
| 3 | 450 | 120 | 15.4 |  | 58.20 |  | 55.3430 | 56.4720 | |  | 4.91 | 2.969 |
| 4 | 550 | 120 | 15.4 |  | 235.3 |  | 235.294 | 235.949 | |  | 0 | -0.276 |
| 5 | 450 | 60 | 52.6 |  | 20.70 |  | 18.4150 | 19.4940 | |  | 11.04 | 5.826 |
| 6 | 550 | 60 | 52.6 |  | 172.01 |  | 170.721 | 171.435 | |  | 0.75 | 0.334 |
| 7 | 450 | 120 | 52.6 |  | 42.30 |  | 41.1750 | 42.2730 | |  | 2.66 | 0.064 |
| 8 | 550 | 120 | 52.6 |  | 206.7 |  | 207.604 | 208.660 | |  | -0.44 | -0.948 |
| 9 | 450 | 60 | 15.4 |  | 26.90 |  | 26.6050 | 27.4730 | |  | 1.1 | -2.13 |
| 10 | 550 | 60 | 15.4 |  | 193.8 |  | 192.434 | 191.709 | |  | 0.7 | 1.079 |
| 11 | 450 | 120 | 15.4 |  | 54.80 |  | 55.3430 | 56.4720 | |  | -0.99 | -3.051 |
| 12 | 550 | 120 | 15.4 |  | 237.4 |  | 235.294 | 235.949 | |  | 0.89 | 0.611 |
| 13 | 450 | 60 | 52.6 |  | 18.30 |  | 18.4150 | 19.4940 | |  | -0.63 | -6.525 |
| 14 | 550 | 60 | 52.6 |  | 171.4 |  | 170.721 | 171.435 | |  | 0.4 | -0.02 |
| 15 | 450 | 120 | 52.6 |  | 43.70 |  | 41.1750 | 42.2730 | |  | 5.78 | 3.265 |
| 16 | 550 | 120 | 52.6 |  | 210.6 |  | 207.604 | 208.660 | |  | 1.42 | 0.921 |
| 17 | 500 | 90 | 34.0 |  | 154.2 |  | 152.650 | 153.165 | |  | 1.01 | 0.671 |
| 18 | 500 | 90 | 34.0 |  | 152.93 |  | 152.650 | 153.165 | |  | 0.18 | -0.154 |
| 19 | 500 | 90 | 34.0 |  | 155.3 |  | 152.650 | 153.165 | |  | 1.71 | 1.375 |
| 20 | 450 | 90 | 34.0 |  | 33.01 |  | 35.3840 | 33.1590 | |  | -7.19 | -0.451 |
| 21 | 550 | 90 | 34.0 |  | 202.66 |  | 201.513 | 202.620 | |  | 0.57 | 0.02 |
| 22 | 500 | 60 | 34.0 |  | 135.0 |  | 136.245 | 134.982 | |  | -0.92 | 0.013 |
| 23 | 500 | 120 | 34.0 |  | 164.6 |  | 169.055 | 164.595 | |  | -2.71 | 0.003 |
| 24 | 500 | 90 | 15.4 |  | 161.4 |  | 161.620 | 161.966 | |  | -0.14 | -0.351 |
| 25 | 500 | 90 | 52.6 |  | 140.83 |  | 143.680 | 140.782 | |  | -2.02 | 0.034 |
| 26 | 500 | 90 | 34.0 |  | 151.24 |  | 152.650 | 153.165 | |  | -0.93 | -1.273 |
| 27 | 500 | 90 | 34.0 |  | 154.97 |  | 152.650 | 153.165 | |  | 1.5 | 1.165 |
